# Supplementary material for: Two-dimensional molybdenum carbide 2D-Mo2C as a superior catalyst for CO2 hydrogenation
Source: Nat Commun. 2021 Sep 17;12:5510. doi: 10.1038/s41467-021-25784-0 (PMC8448824; doi:10.1038/s41467-021-25784-0)
Supplement: Supplementary file 1 — Supplementary Information [file 41467_2021_25784_MOESM1_ESM.pdf]

Supplementary Information

Two-Dimensional Molybdenum Carbide 2D-Mo_2C as a Superior Catalyst for CO_2 Hydrogenation

Hui Zhou[1,3], Zixuan Chen[1], Evgenia Kountoupi[1], Athanasia Tsoukalou[1], Paula M. Abdala[1],

Pierre Florian[2], Alexey Fedorov[1,*], and Christoph R. Müller[1,*]

[1]Department of Mechanical and Process Engineering, ETH Zürich, CH 8092 Zürich, Switzerland

[2]CNRS, CEMHTI UPR3079, Université d'Orléans, F-45071 Orléans, France

[3]Present address: Department of Energy and Power Engineering, Tsinghua University, Beijing

100084, China

[*]E-mail: fedoroal@ethz.ch; muelchri@ethz.ch

This PDF file includes the following Supplementary Figures and Tables

Supplementary Fig. 1. Rietveld refinement of the XRD pattern of Mo_2Ga_2C (space group *P*63/*mmc*) and Le Bail fitting of the XRD pattern of multilayered Mo_2CT_x (space group *P*63/*mmc*).
Supplementary Fig. 2. Ga 2*p* XPS of Mo_2Ga_2C and multilayered Mo_2CT_x.
Supplementary Fig. 3. Raman spectra of β-Mo_2C, Mo_2Ga_2C, and Mo_2CT_x.
Supplementary Fig. 4. Temperature programmed oxidation (TPO) study of Mo_2CT_x under air (100 mL min^{-1}) in TGA-DSC.
Supplementary Fig. 5. XRD of Mo_2CT_x after oxidation in air (100 mL min^{-1}) at 600 °C in TGA-DSC.
Supplementary Fig. 6. Temperature programmed reduction (TPR) of Mo_2CT_x under 5% H_2/Ar (50 mL min^{-1}, 5 °C min^{-1}).
Supplementary Fig. 7. TPR study of Mo_2CT_x under 5% H_2/N_2 (100 mL min^{-1}) in TGA-DSC.
Supplementary Fig. 8. In situ Raman TPR study of Mo_2CT_x under 100% H_2/Ar (50 mL min^{-1}, 5 °C min^{-1}).

Supplementary Fig. 9. Reduction of Mo_2CT_x followed by in situ XRD.
Supplementary Fig. 10. Le Bail fittings of XRD patterns of 2D-Mo_2C and $Mo_2CT_{x\text{-}700}$.
Supplementary Fig. 11. Mo 3*d* XPS of $Mo_2CT_{x\text{-}300}$.
Supplementary Fig. 12. Fitted Mo states of the prepared materials assessed by Mo 3*d* XPS.
Supplementary Fig. 13. XPS survey of Mo_2CT_x, $Mo_2CT_{x\text{-}300}$, and 2D-Mo_2C.
Supplementary Fig. 14. F 1*s* XPS of Mo_2CT_x and 2D-Mo_2C.
Supplementary Fig. 15. Mo 3*d* XPS of Mo_2CT_x pretreated under 10% H_2 or 100% H_2 at 500 °C for 2 hours.
Supplementary Fig. 16. SEM of Mo_2CT_x.
Supplementary Fig. 17. SEM images of β-Mo_2C.
Supplementary Fig. 18. SEM images of $Mo_2CT_{x\text{-}300}$.
Supplementary Fig. 19. SEM images of 2D-Mo_2C.
Supplementary Fig. 20. STEM of Mo_2CT_x and 2D-Mo_2C.
Supplementary Fig. 21. Selected area electron diffraction (SAED) of 2D-Mo_2C.
Supplementary Fig. 22. Selected area electron diffraction (SAED) of $Mo_2CT_{x\text{-}700}$.
Supplementary Fig. 23. Selected area electron diffraction (SAED) of β-Mo_2C.
Supplementary Fig. 24. Mo K-edge XANES without exposure to air.
Supplementary Fig. 25. Fourier-transform of the k^3-weighted EXAFS of Mo_2CT_x, $Mo_2CT_{x\text{-}300}$, and 2D-Mo_2C.
Supplementary Fig. 26. Fitting of the k^3-weighted EXAFS data of the different catalysts.
Supplementary Fig. 27. CO temperature-programmed desorption (TPD) of Mo_2CT_x and 2D-Mo_2C.
Supplementary Fig. 28. H_2 TPD of β-Mo_2C and 2D-Mo_2C.
Supplementary Fig. 29. Catalytic performance of Mo_2CT_x with different pretreatments.
Supplementary Fig. 30. Catalytic performance of β-Mo_2C.
Supplementary Fig. 31. NH_3 temperature programmed desorption of $Mo_2CT_{x\text{-TOS1h}}$, 2D-Mo_2C, and β-Mo_2C.
Supplementary Fig. 32. WGS catalytic activity of Mo_2CT_x and 2D-Mo_2C.
Supplementary Fig. 33. Mo K-edge XANES of 2D-Mo_2C before and after 10-hour TOS of WGS reaction (500 °C, 1 bar, $CO/H_2O/N_2$ = 1/1/9, contact time 0.03 s g mL^{-1}).
Supplementary Fig. 34. Intrinsic CO_2 reaction rate and product formation rates normalized per mass of surface Mo sites (denoted as Mo(surf)).
Supplementary Fig. 35. Catalytic performance of Mo_2CT_x reduced at 500 °C for 2 hours under 100% H_2 (2D-Mo_2C) or 10% H_2/N_2.
Supplementary Fig. 36. Thermodynamic equilibrium calculation of CO_2 hydrogenation at different reaction conditions.
Supplementary Fig. 37. Catalytic performance of Mo_2CT_x at different temperatures.
Supplementary Fig. 38. Catalytic performance of 2D-Mo_2C at different temperatures.
Supplementary Fig. 39. Intrinsic selectivity at different reaction temperatures.
Supplementary Fig. 40. C_2–C_5 hydrocarbons generated from CO_2 hydrogenation using a 2D-Mo_2C catalyst (330 °C, 25 bar, $H_2/CO_2/N_2$ = 3/1/1, contact time 0.03 s g mL^{-1})
Supplementary Fig. 41. Catalytic performance of Mo_2CT_x at different pressures.
Supplementary Fig. 42. Catalytic performance of 2D-Mo_2C at different pressures.
Supplementary Fig. 43. Intrinsic selectivity at different reaction pressures.
Supplementary Fig. 44. Catalytic performance of Mo_2CT_x with different H_2/CO_2 ratios.
Supplementary Fig. 45. Catalytic performance of 2D-Mo_2C with different H_2/CO_2 ratios.

Supplementary Fig. 46. Intrinsic selectivity with different H_2/CO_2 ratios.
Supplementary Fig. 47. Reaction at 430 °C and 1 bar for high CO selectivity with Mo_2CT_x.
Supplementary Fig. 48. Comparison of CO formation rate and CO yield with 2D-Mo_2C, Cu-ZnO-Al_2O_3, and β-Mo_2C.
Supplementary Fig. 49. Comparison of CH_4 formation for 2D-Mo_2C, β-Mo_2C, and Cu-ZnO-Al_2O_3.
Supplementary Fig. 50. Stability of Mo_2CT_x during CO_2 hydrogenation reaction at standard conditions (230 °C, 25 bar, $H_2/CO_2/N_2$ = 3/1/1, contact time 0.03 s g mL^{-1}) after 36 hours of TOS at 230 and 430 °C, respectively.
Supplementary Fig. 51. Le Bail fitting of the XRD pattern of Mo_2CT_x after 36 hours of TOS at 430 °C.
Supplementary Fig. 52. Mo K-edge XAS of Mo_2CT_x before and after 36 hours of TOS at 430 °C.
Supplementary Fig. 53. Mo 3*d* XPS of Mo_2CT_x before and after 36 hours of TOS at 430 °C.
Supplementary Fig. 54. Comparison of $Mo_2CT_{x\text{-TOS36h(430)-500}}$ and 2D-Mo_2C.
Supplementary Fig. 55. Le Bail fitting of the XRD pattern of 2D-Mo_2C after 36 hours of TOS at 430 °C.
Supplementary Fig. 56. Operando Raman spectroscopy at CO_2 hydrogenation conditions with 2D-Mo_2C.
Supplementary Fig. 57. Mo K-edge XAS of 2D-Mo_2C before and after 36 hours of TOS at 430 °C.
Supplementary Fig. 58. Chemical looping type operation of CO_2 hydrogenation studied by TGA.
Supplementary Fig. 59. CO_2 dissociation study on β-Mo_2C (430 °C, 1 bar).
Supplementary Fig. 60. ^{13}C NMR of the 2D-Mo_2C after exposure to the reaction stream at 430 °C for 9 hours and the fresh Mo_2CT_x.
Supplementary Fig. 61. ^{95}Mo NMR of the 2D-Mo_2C after exposure to the reaction stream at 430 °C for 9 hours and the fresh Mo_2CT_x.
Supplementary Fig. 62. C 1*s* XPS of the $Mo2CT_{x\text{-300}}$ collected using an air-tight transfer cell.
Supplementary Fig. 63. The (C–O+COO)/C–Mo ratio from C 1*s* XPS of the different catalysts assessed.

Supplementary Table 1. XPS fitting parameters of Mo 3*d* for fresh and used (TOS = 36 hours) catalysts.
Supplementary Table 2. Mo K-edge EXAFS fitting of fresh and used (TOS = 36 hours) catalysts.
Supplementary Table 3. Intrinsic activity of the catalysts for CO_2 hydrogenation (230 °C, 25 bar, $H_2/CO_2/N_2$ = 3/1/1).
Supplementary Table 4. CO generation from the CO_2 dissociation experiment.

Supplementary Figures

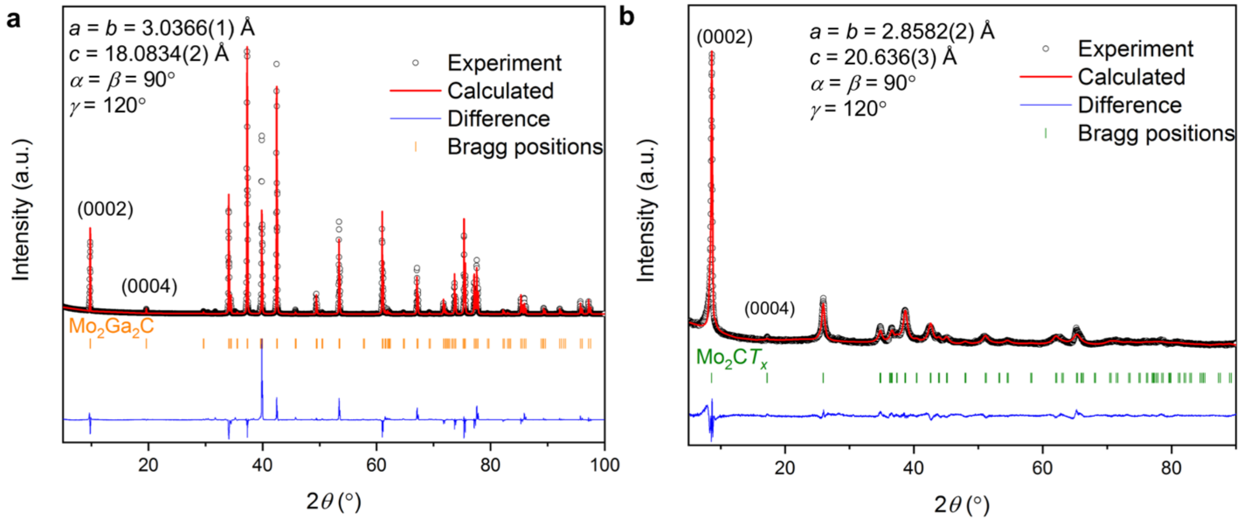

Supplementary Fig. 1. **a** Rietveld refinement of the XRD pattern of Mo_2Ga_2C (space group *P*63/*mmc*). **b** Le Bail fitting of the XRD pattern of multilayered Mo_2CT_x (space group *P*63/*mmc*).

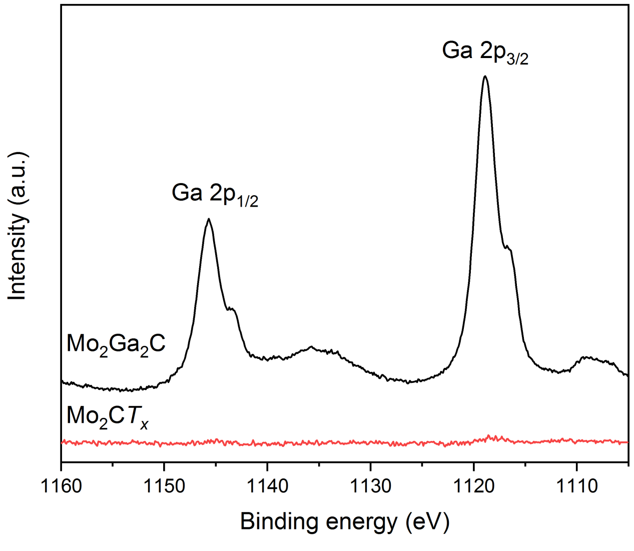

Supplementary Fig. 2. Ga 2*p* XPS of Mo_2Ga_2C and multilayered Mo_2CT_x.

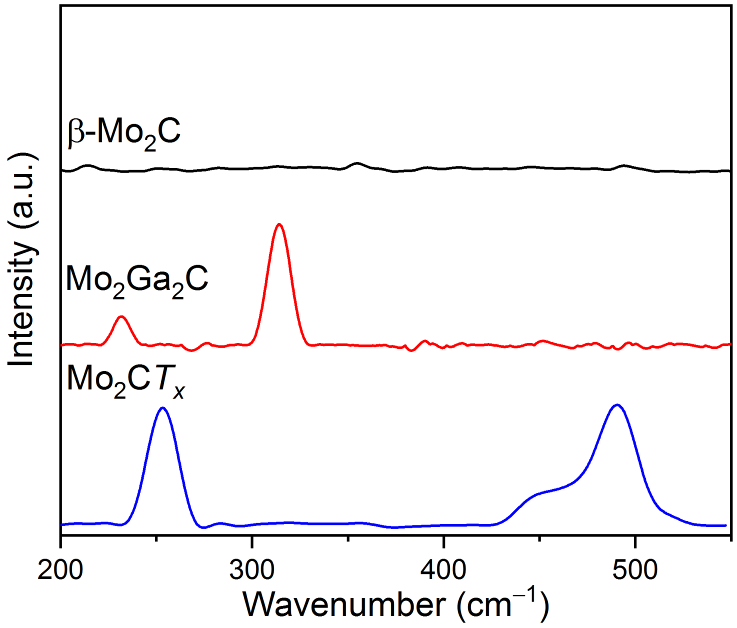

Supplementary Fig. 3. Raman spectra of β-Mo_2C, Mo_2Ga_2C, and Mo_2CT_x.

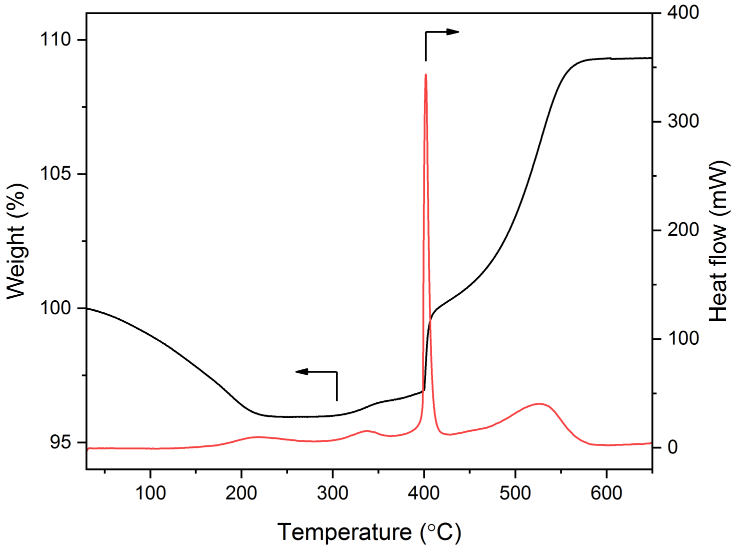

Supplementary Fig. 4. Temperature programmed oxidation (TPO) study of Mo_2CT_x under air (100 mL min^{-1}) in TGA-DSC.

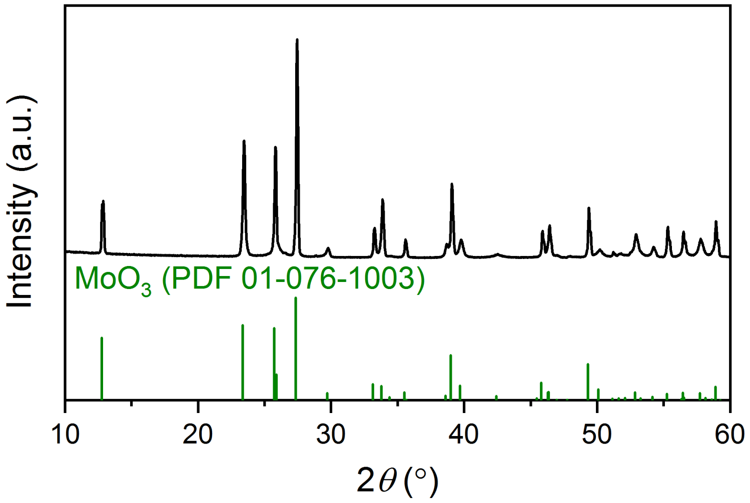

Supplementary Fig. 5. XRD of Mo_2CT_x after oxidation in air (100 mL min^{-1}) at 600 °C in TGA-DSC.

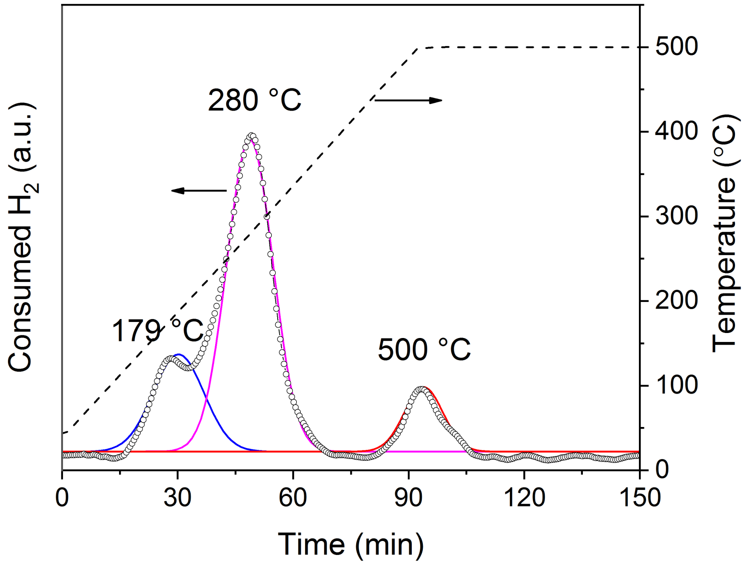

Supplementary Fig. 6. Temperature programmed reduction (TPR) of Mo_2CT_x under 5% H_2/Ar (50 mL min^{-1}, 5 °C min^{-1}).

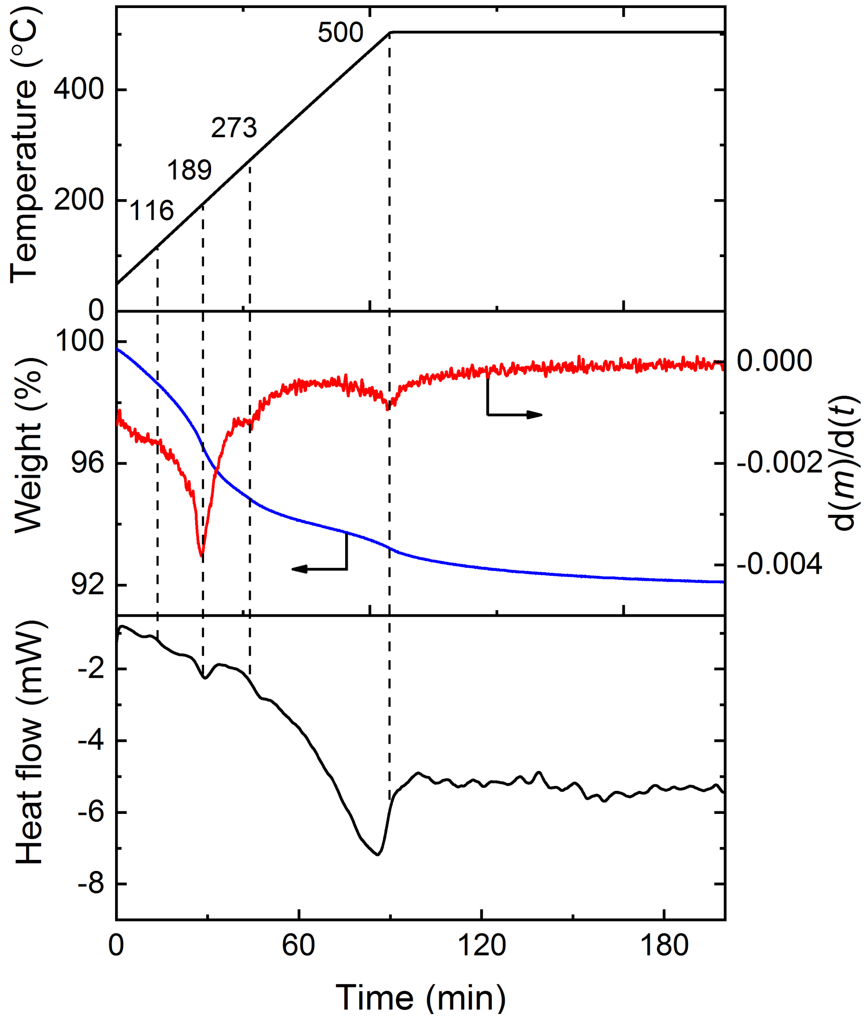

Supplementary Fig. 7. TPR study of Mo_2CT_x under 5% H_2/N_2 (100 mL min^{-1}) in TGA-DSC.

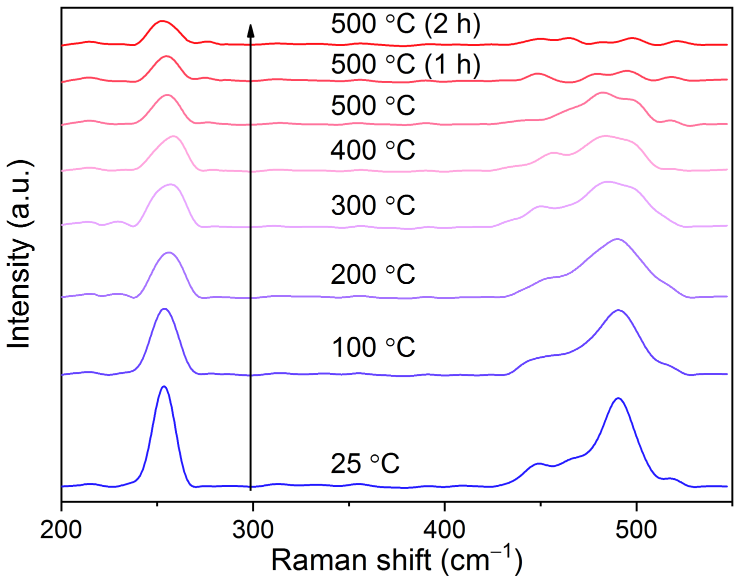

Supplementary Fig. 8. In situ Raman TPR study of Mo_2CT_x under 100% H_2/Ar (50 mL min^{-1}, 5 °C min^{-1}).

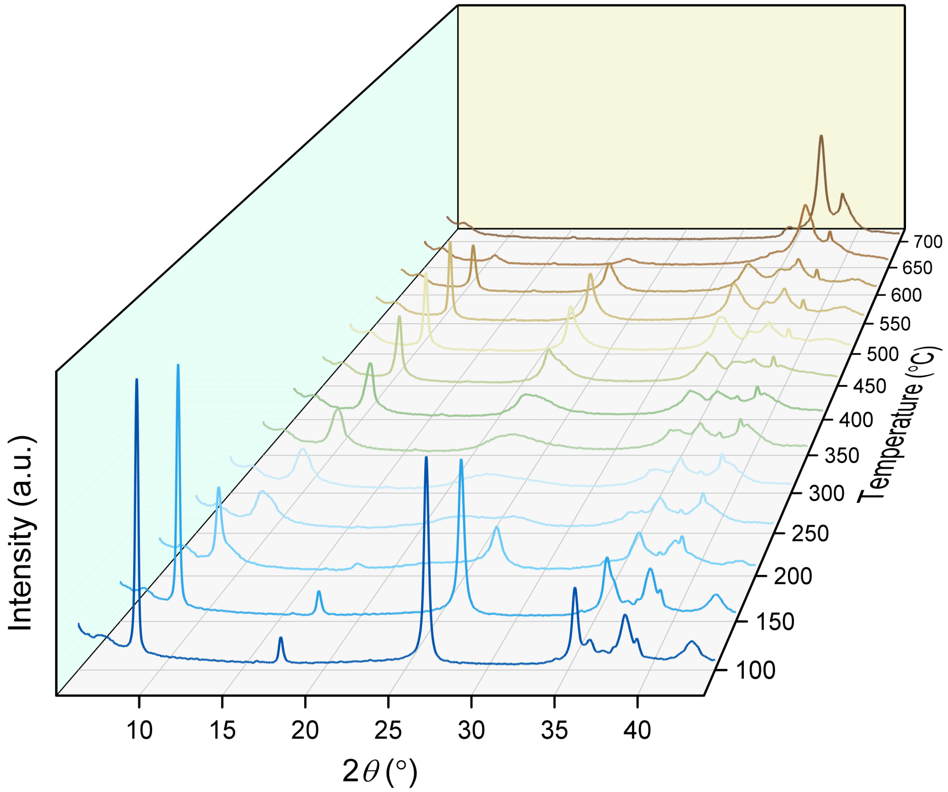

Supplementary Fig. 9. Reduction of Mo_2CT_x followed by in situ XRD.

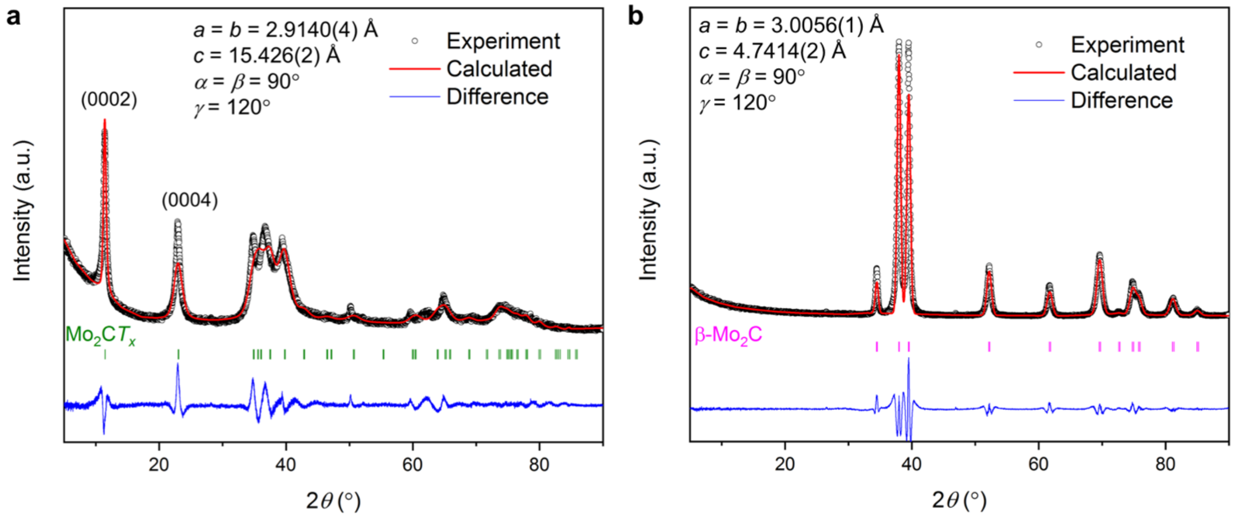

Supplementary Fig. 10. **a** Le Bail fittings of XRD patterns of 2D-Mo_2C and **b** $Mo_2CT_{x\text{-}700}$.

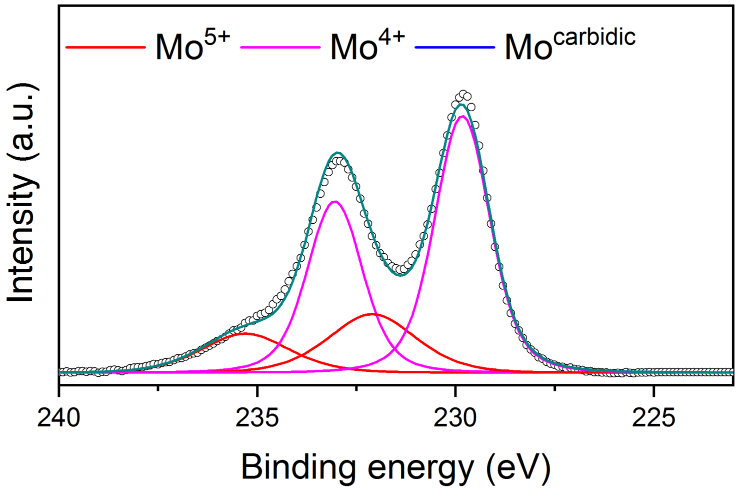

Supplementary Fig. 11. Mo 3*d* XPS of $Mo_2CT_{x\text{-}300}$ (see Supplementary Table 1 for the fitting parameters).

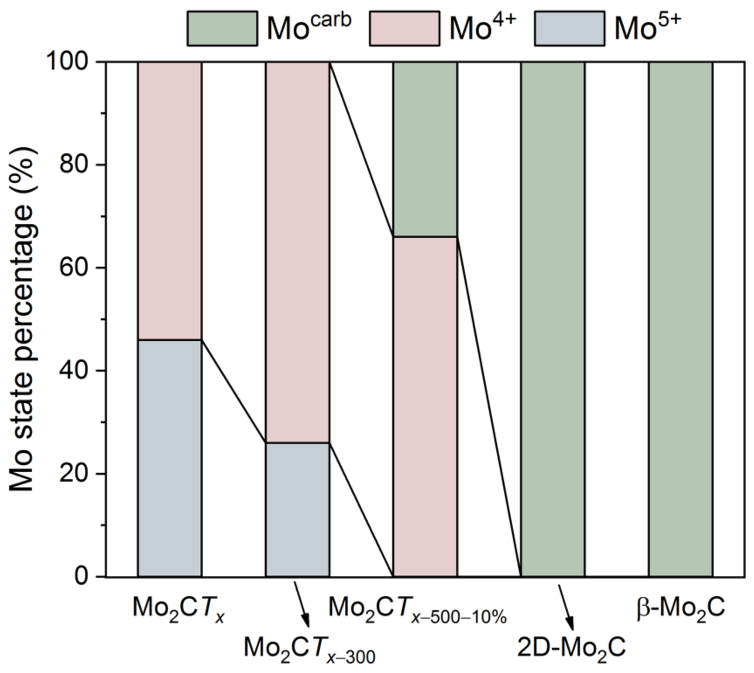

Supplementary Fig. 12. Fitted Mo states of the prepared materials assessed by Mo 3*d* XPS.

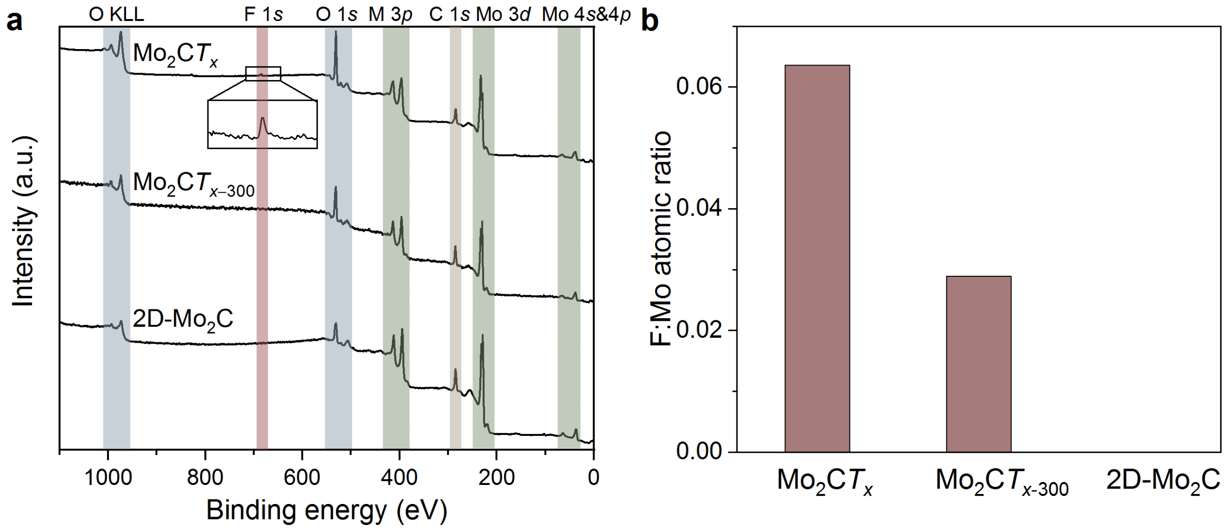

Supplementary Fig. 13. XPS survey of Mo_2CT_x, $Mo_2CT_{x\text{-}300}$, and 2D-Mo_2C. **a** XPS survey spectra. **b** F/Mo atomic ratio as obtained from the XPS survey.

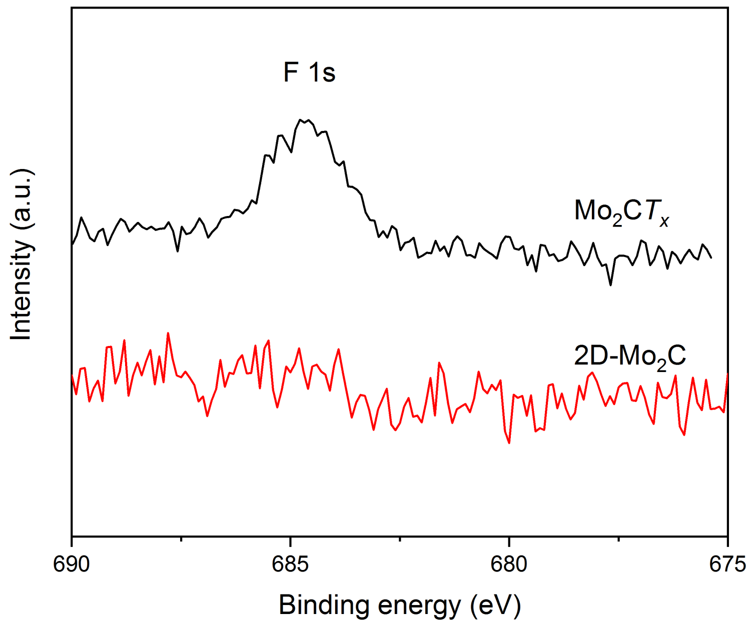

Supplementary Fig. 14. F 1*s* XPS of Mo_2CT_x and 2D-Mo_2C.

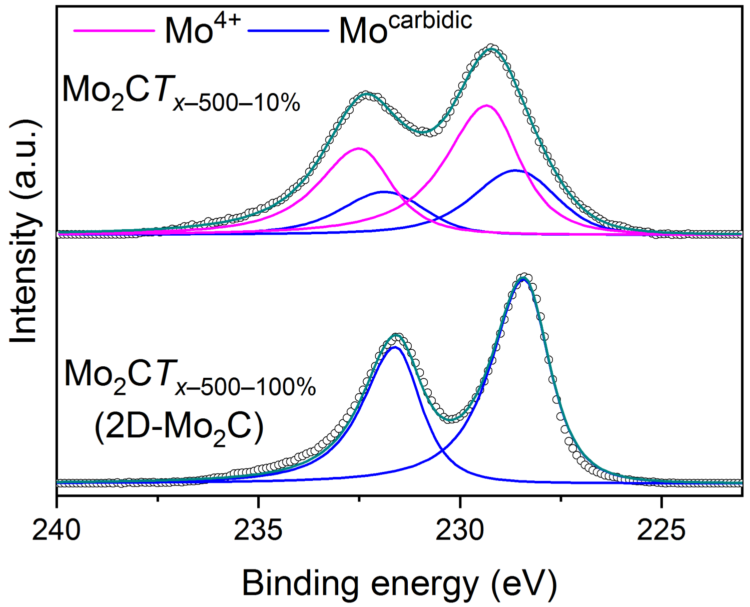

Supplementary Fig. 15. Mo 3*d* XPS of Mo_2CT_x pretreated under 10% H_2 or 100% H_2 at 500 °C for 2 hours.

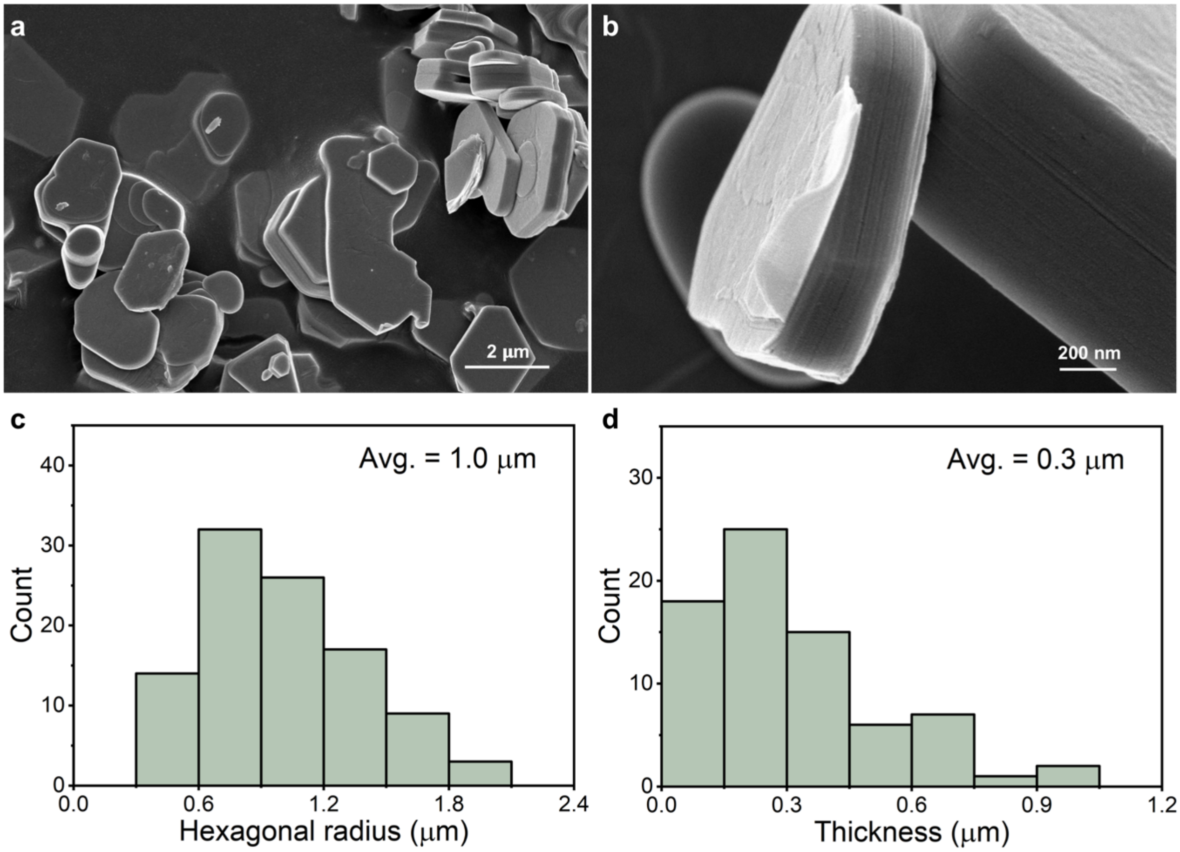

Supplementary Fig. 16. SEM of Mo_2CT_x. **a** and **b** SEM images. **c** Histogram of circumscribing radius of the hexagons. **d** Histogram of thickness of the hexagons.

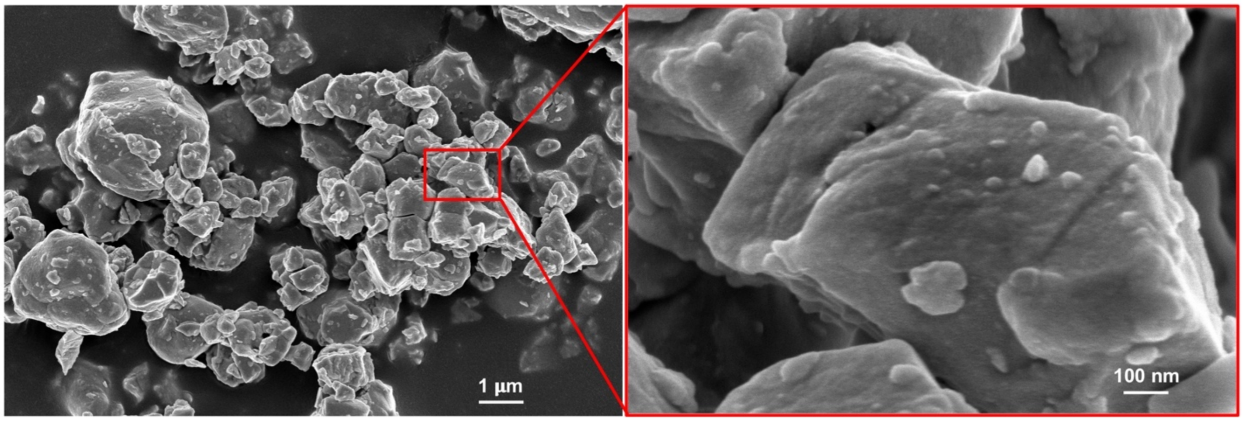

Supplementary Fig. 17. SEM images of β-Mo_2C.

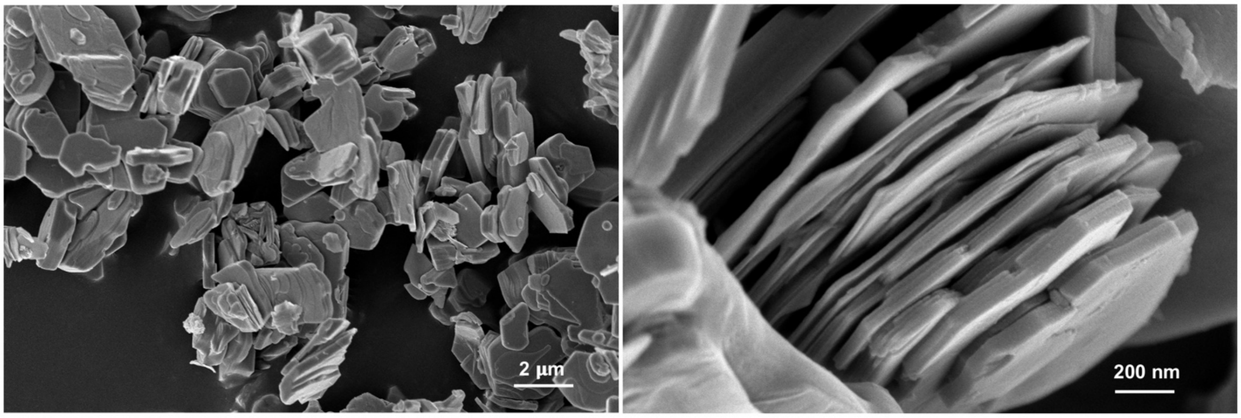

Supplementary Fig. 18. SEM images of $Mo_2CT_{x\text{-}300}$.

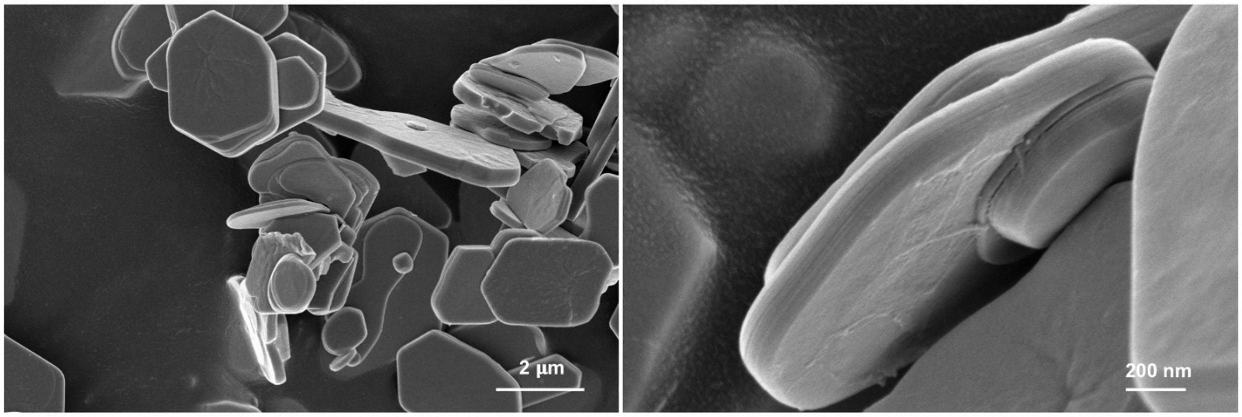

Supplementary Fig. 19. SEM images of 2D-Mo_2C.

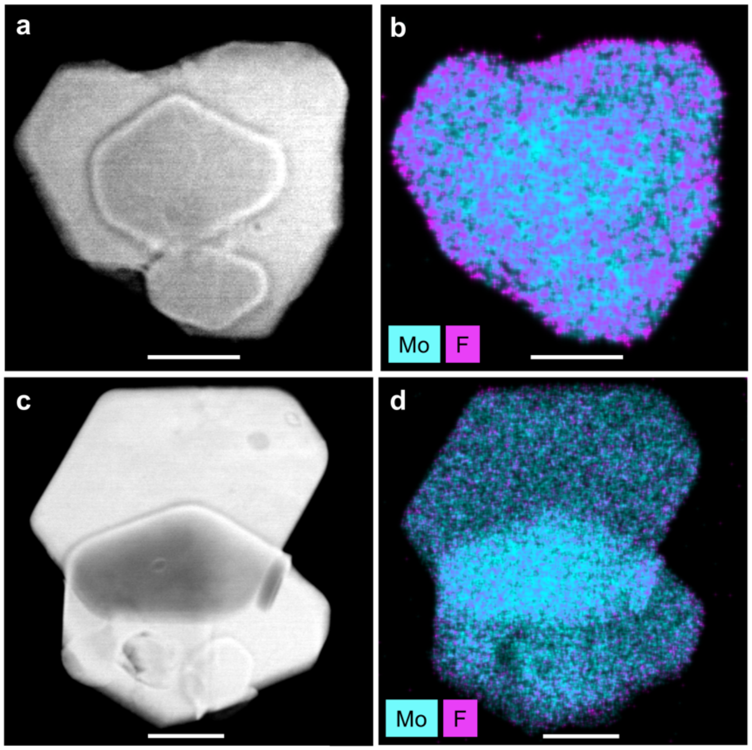

Supplementary Fig. 20. STEM of Mo_2CT_x and 2D-Mo_2C. **a** HAADF-STEM of Mo_2CT_x. **b** EDX of Mo_2CT_x. **c** HAADF-STEM of 2D-Mo_2C. **d** EDX of 2D-Mo_2C. Scale bar: 1 µm.

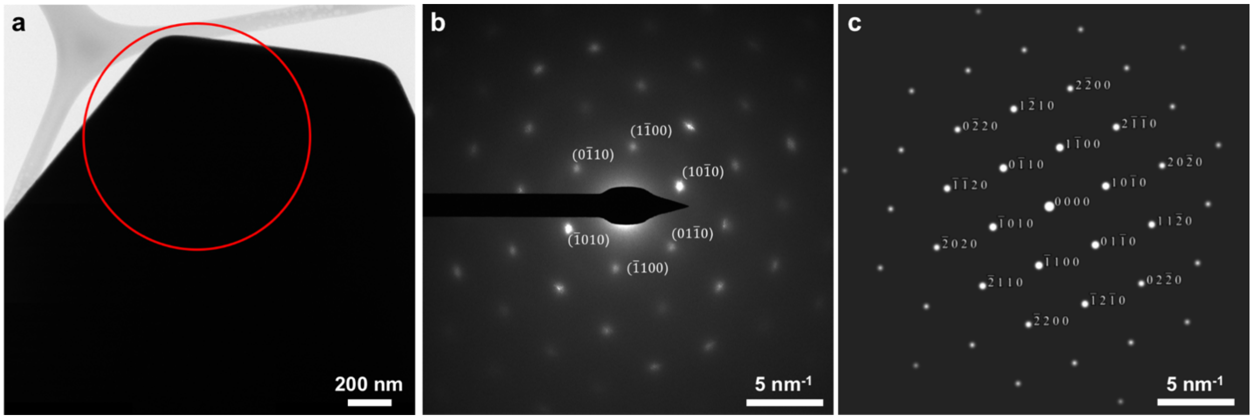

Supplementary Fig. 21. Selected area electron diffraction (SAED) of 2D-Mo_2C. **a** SAED region. **b** Measured SAED pattern. **c** Simulated SAED pattern. The diffraction pattern is taken along the [0001] direction.

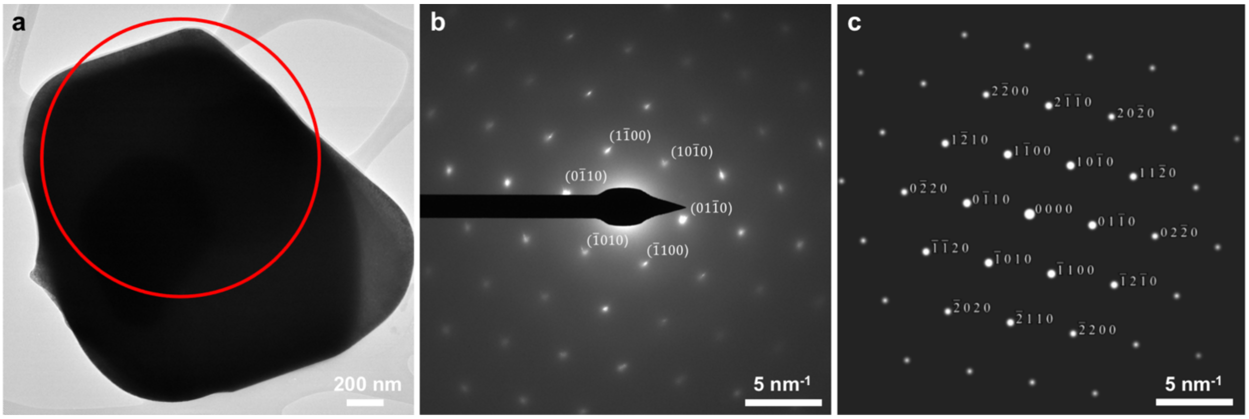

Supplementary Fig. 22. Selected area electron diffraction (SAED) of $Mo_2CT_{x\text{-}700}$. **a** SAED region. **b** Measured SAED pattern. **c** Simulated SAED pattern. The diffraction pattern is taken along the [0001] direction.

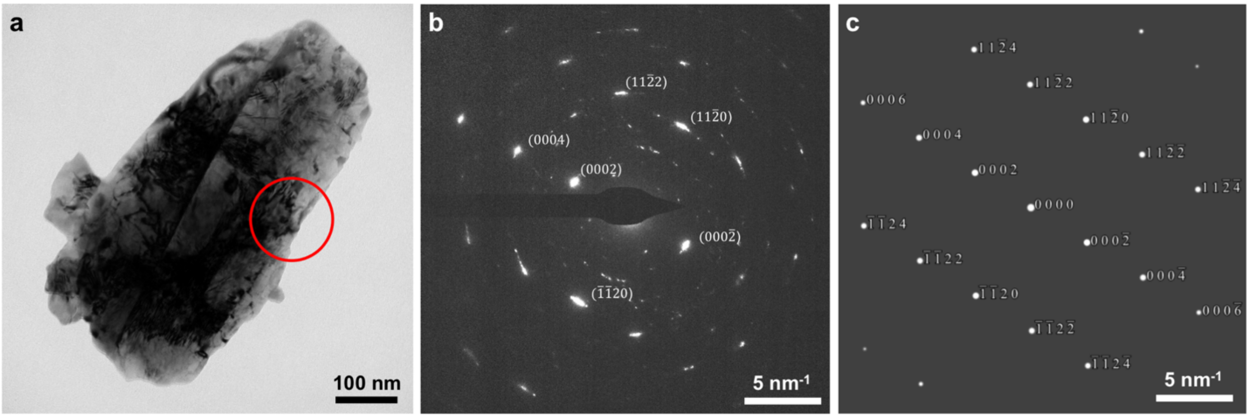

Supplementary Fig. 23. Selected area electron diffraction (SAED) of β-Mo_2C. **a** SAED region. **b** Measured SAED pattern. **c** Simulated SAED pattern. The diffraction pattern is taken along the $[\bar{1}100]$ direction.

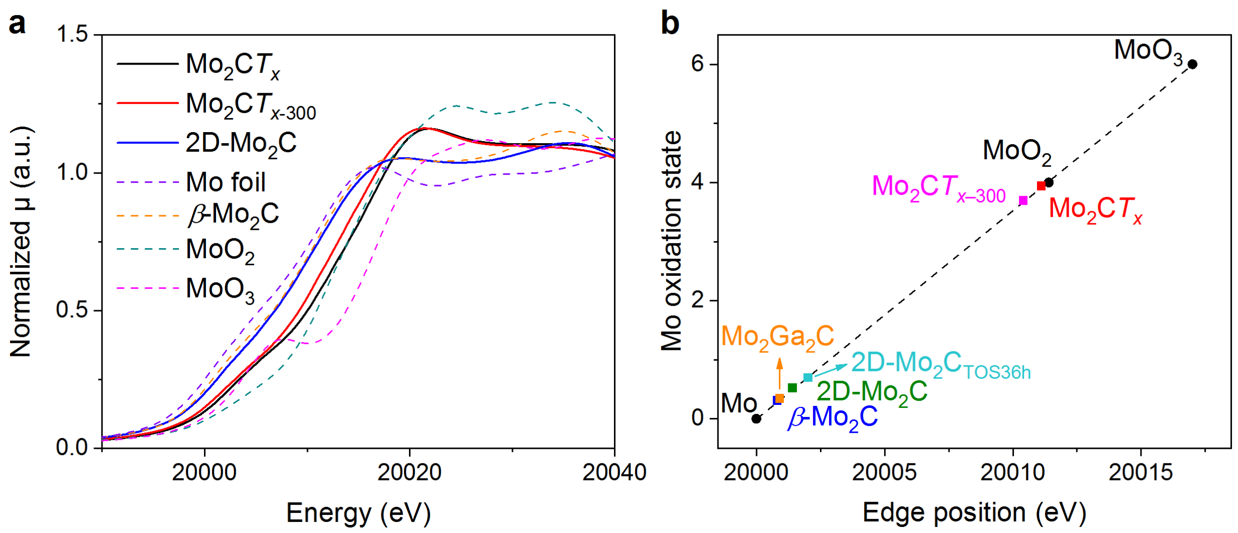

Supplementary Fig. 24. Mo K-edge XANES without exposure to air. **a** Mo XANES. **b** Mo oxidation state determined from the edge position in the XANES spectra. The edge position is defined here as the first inflection point of the XANES spectra after the pre-edge feature (where the pre-edge corresponds to the forbidden 1s-4d transition)[1].

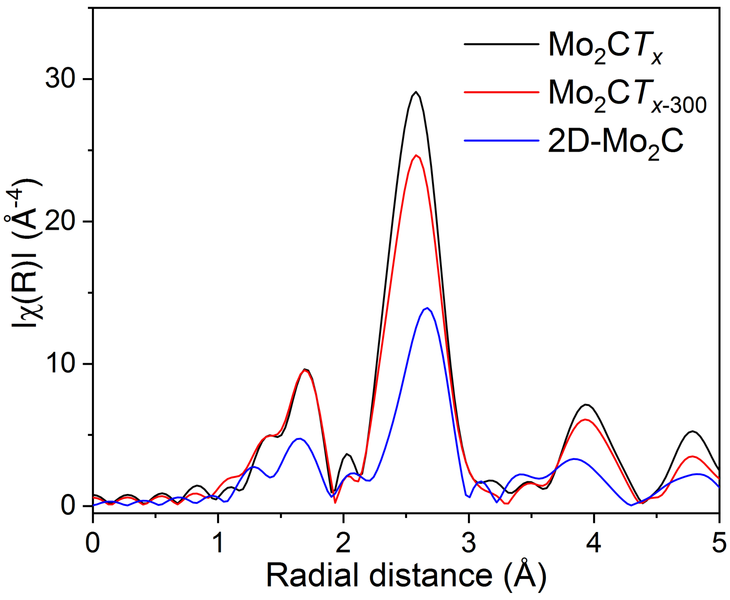

Supplementary Fig. 25. Fourier-transform of the k^3-weighted EXAFS of Mo_2CT_x, $Mo_2CT_{x\text{-}300}$, and 2D-Mo_2C.

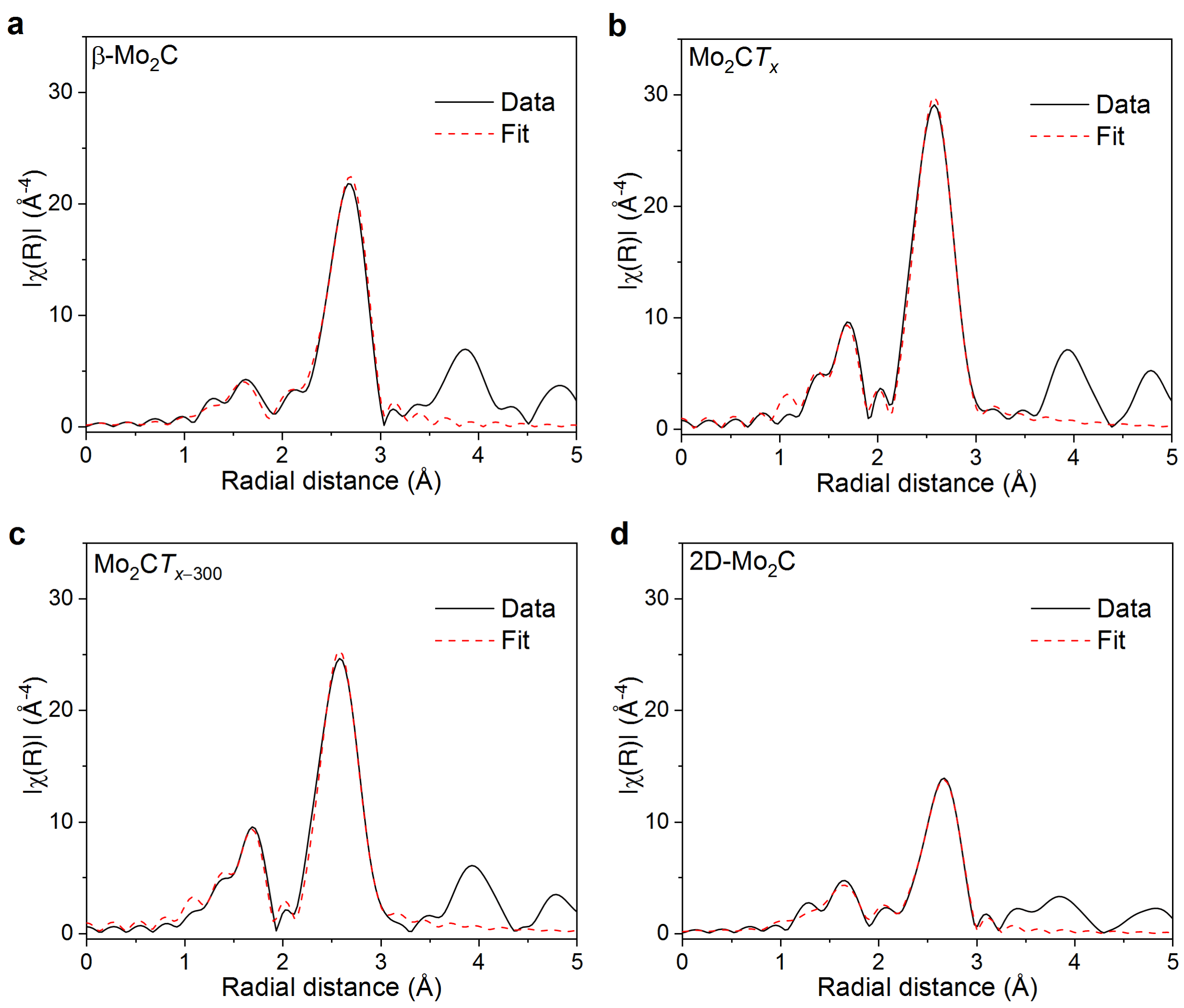

Supplementary Fig. 26. Fitting of the k^3-weighted EXAFS data of the different catalysts. **a** β-Mo_2C. **b** Mo_2CT_x. **c** $Mo_2CT_{x\text{-}300}$. **d** 2D-Mo_2C.

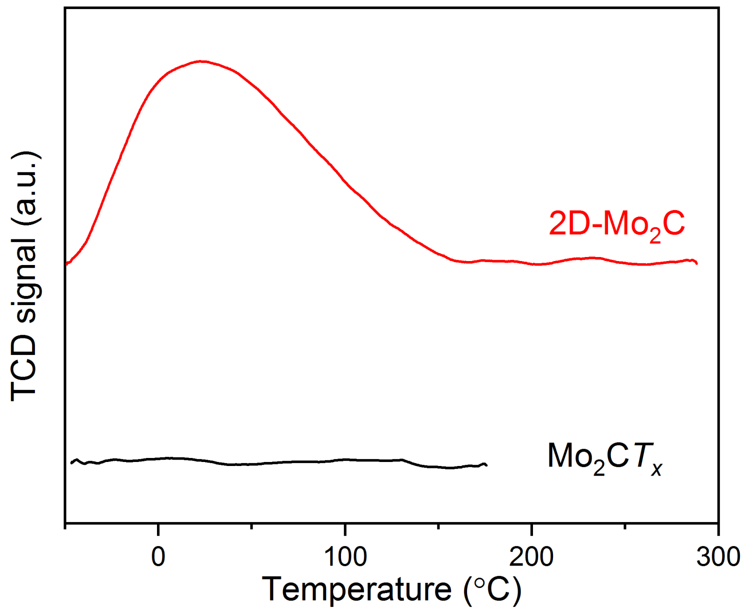

Supplementary Fig. 27. CO temperature-programmed desorption (TPD) of Mo_2CT_x and 2D-Mo_2C.

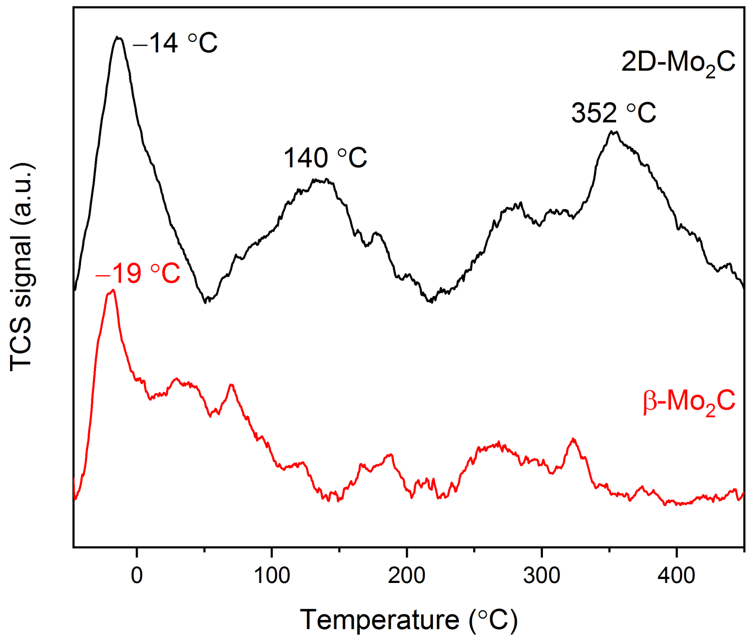

Supplementary Fig. 28. H_2 TPD of β-Mo_2C and 2D-Mo_2C.

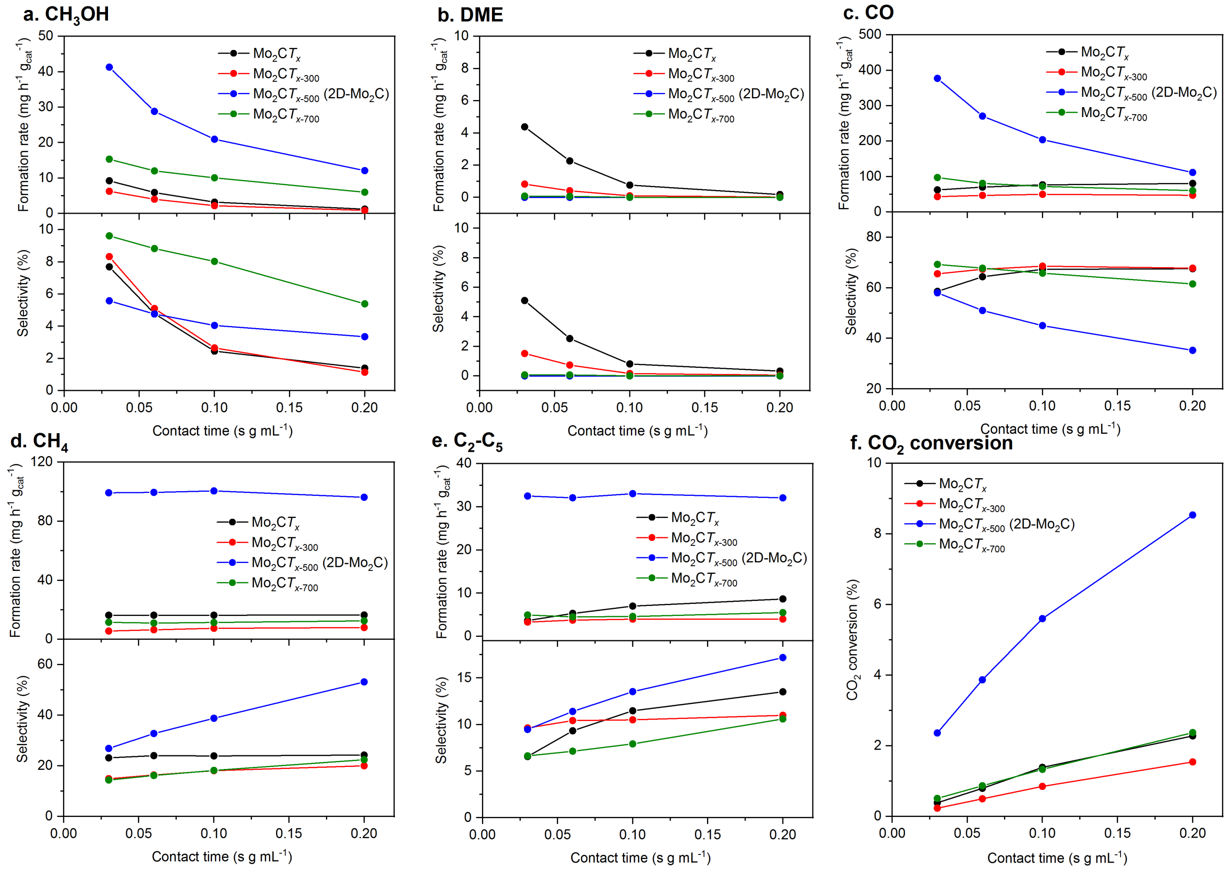

Supplementary Fig. 29. Catalytic performance of Mo_2CT_x with different pretreatments. Reaction conditions: 230 °C, 25 bar, $H_2/CO_2/N_2$ = 3/1/1.

Note that the lines in Supplementary Figures 29, 30, 37, 38, 41, 42, 44, 45, and 47-49 are added to guide the eye.

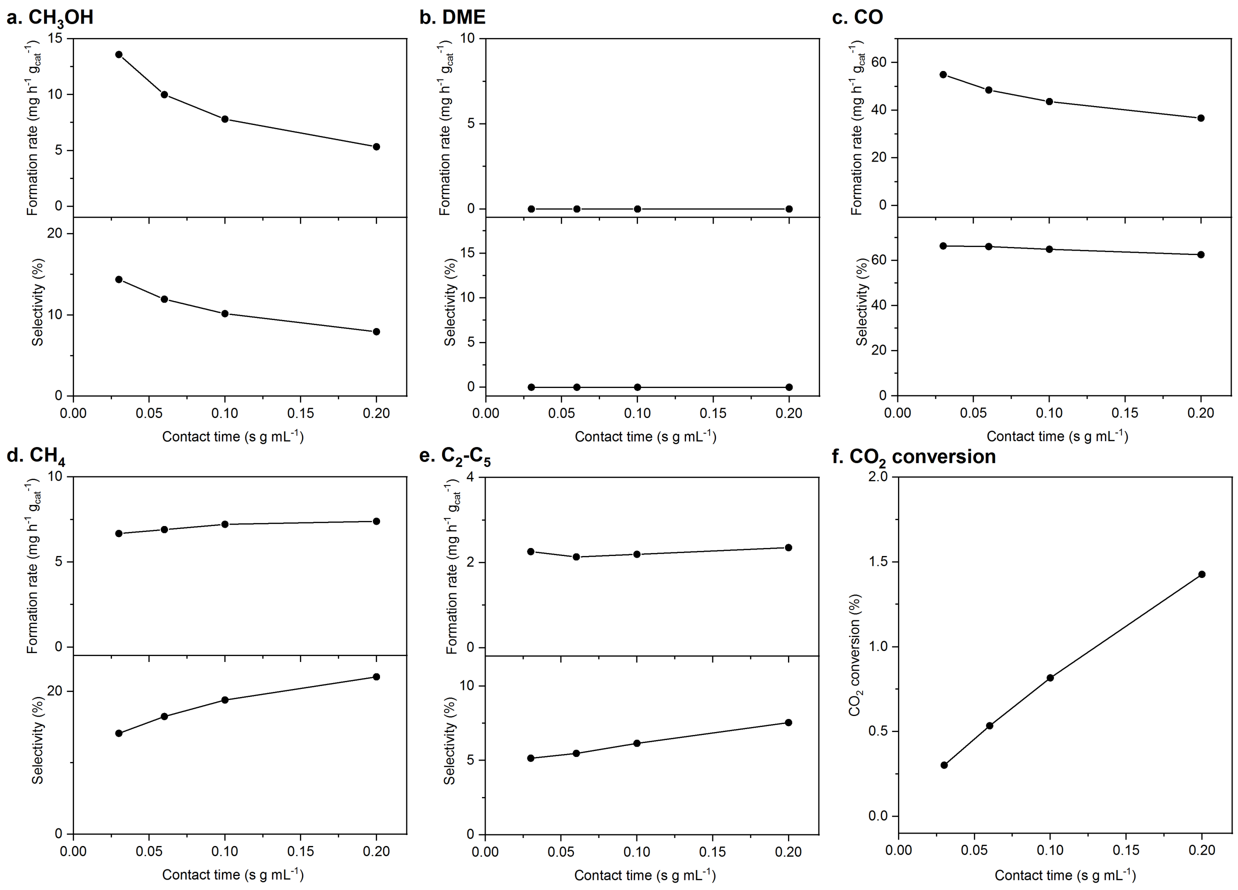

Supplementary Fig. 30. Catalytic performance of β-Mo_2C. Reaction conditions: 230 °C, 25 bar, $H_2/CO_2/N_2$ = 3/1/1.

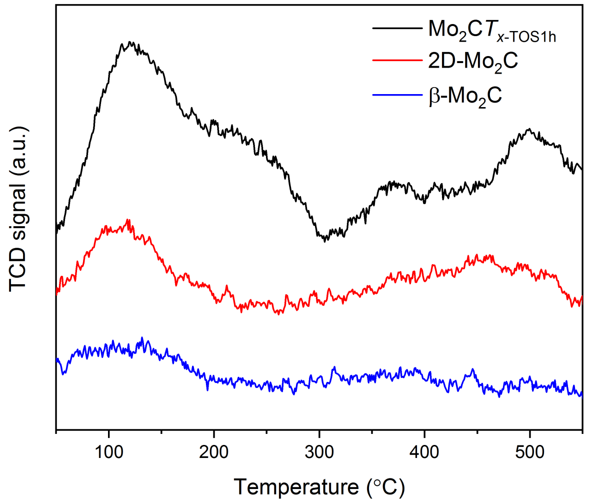

Supplementary Fig. 31. NH_3 temperature programmed desorption of $Mo_2CT_{x\text{-TOS1h}}$, 2D-Mo_2C, and β-Mo_2C.

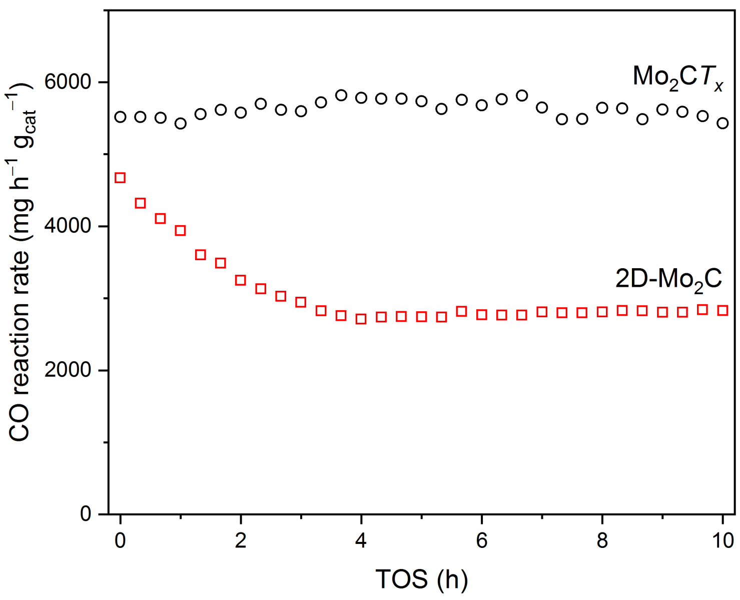

Supplementary Fig. 32. WGS catalytic activity of Mo_2CT_x and 2D-Mo_2C. Reaction conditions: 500 °C, 1 bar, $CO/H_2O/N_2$ = 1/1/9, contact time 0.03 s g_{cat} mL^{-1}.

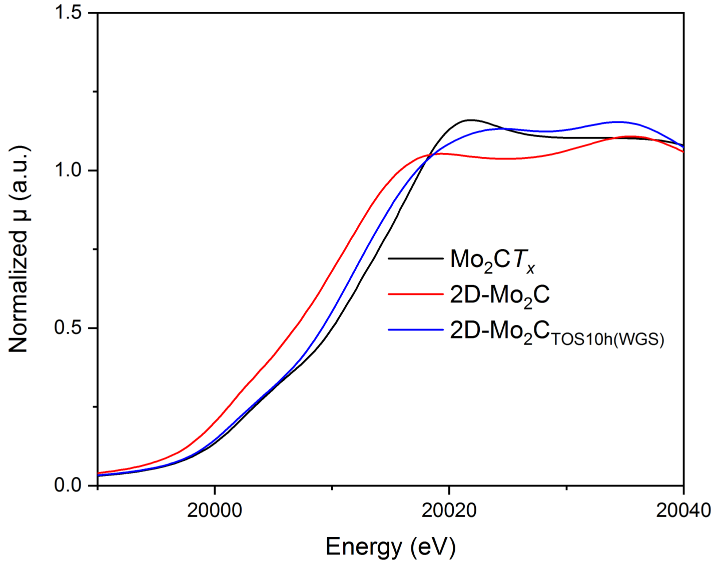

Supplementary Fig. 33. Mo K-edge XANES of 2D-Mo_2C before and after 10-hour TOS of WGS reaction (500 °C, 1 bar, $CO/H_2O/N_2$ = 1/1/9, contact time 0.03 s g_{cat} mL^{-1}).

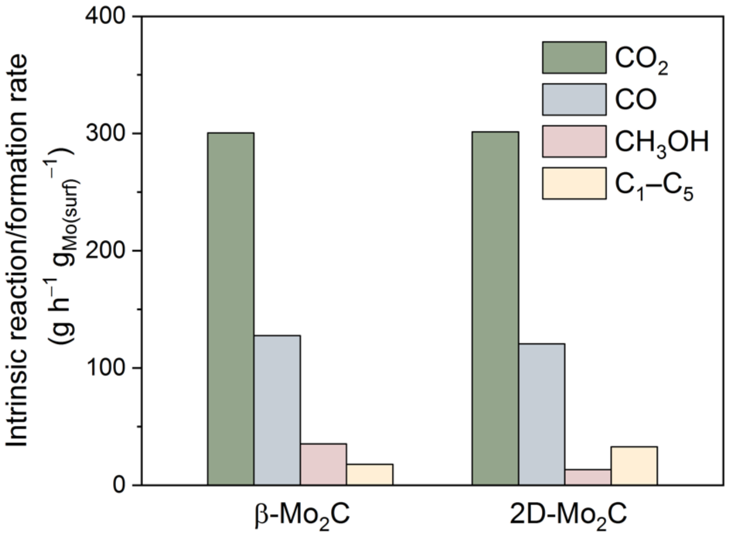

Supplementary Fig. 34. Intrinsic CO_2 reaction rate and product formation rates normalized per mass of surface Mo sites (denoted as Mo(surf)). Reaction conditions: 230 °C, 25 bar, $H_2/CO_2/N_2$ = 3/1/1. The surface Mo sites are determined by CO chemisorption (Table 1).

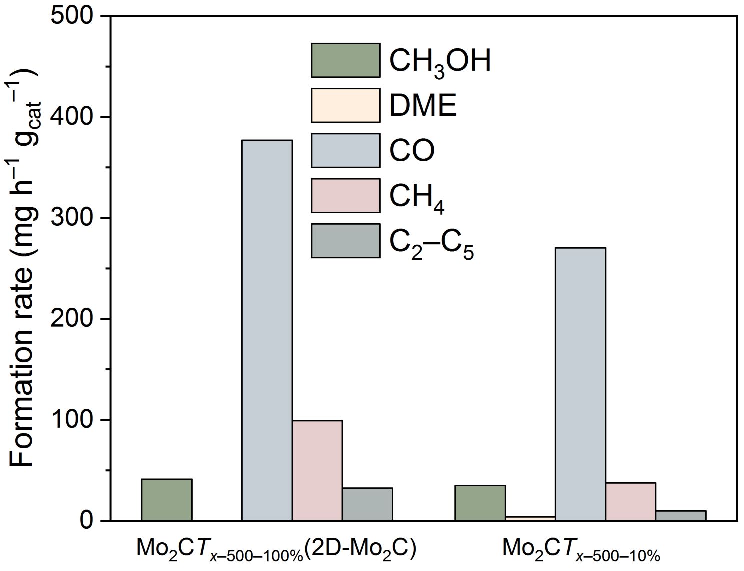

Supplementary Fig. 35. Catalytic performance of Mo_2CT_x reduced at 500 °C for 2 hours under 100% H_2 (2D-Mo_2C) or 10% H_2/N_2. Reaction conditions: 230 °C, 25 bar, $H_2/CO_2/N_2$ = 3/1/1, contact time 0.03 s g_{cat} mL^{-1}.

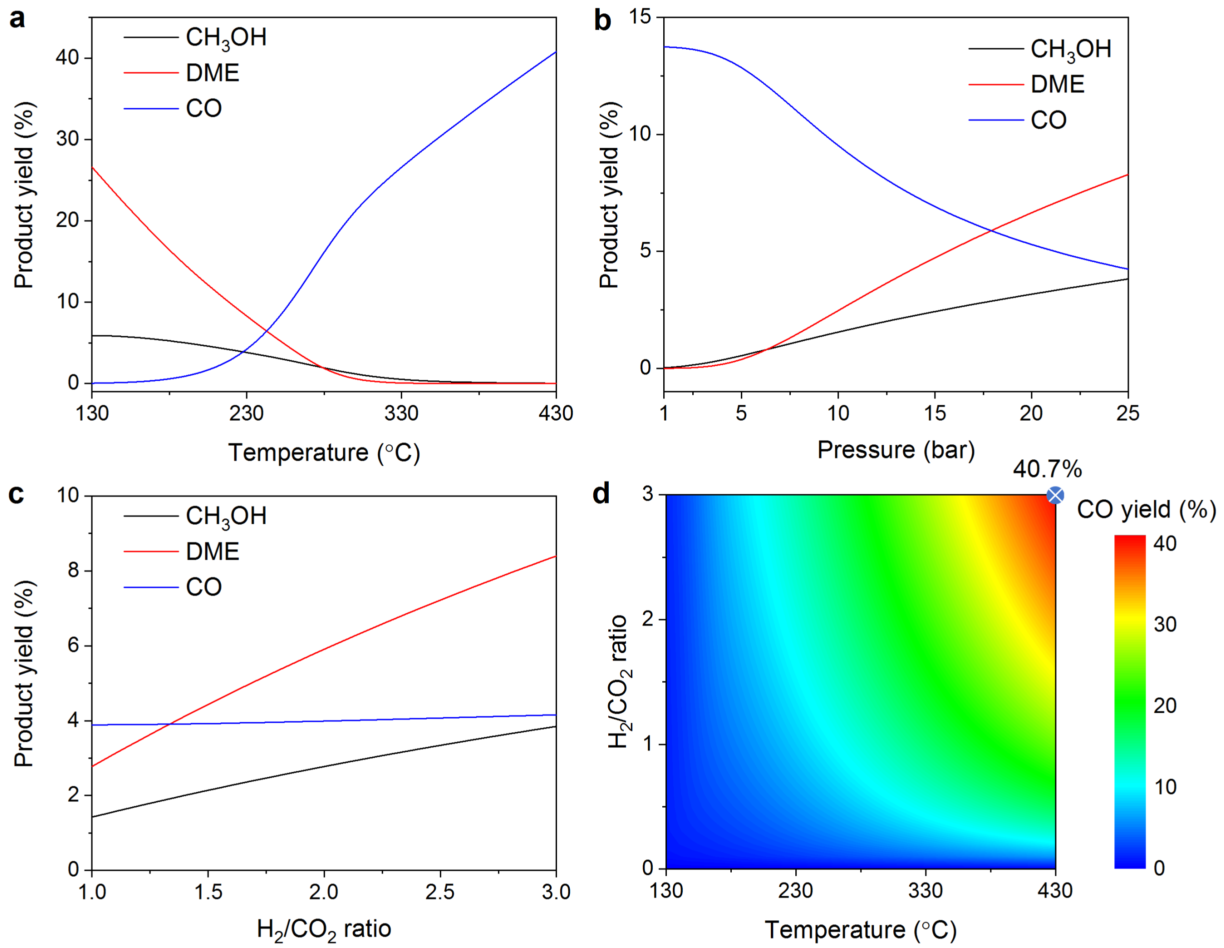

Supplementary Fig. 36. Thermodynamic equilibrium calculation of CO_2 hydrogenation at different reaction conditions. **a** Influence of temperature (25 bar, $H_2/CO_2/N_2$ = 3/1/1). **b** Influence of pressure (230 °C, $H_2/CO_2/N_2$ = 3/1/1). **c** Influence of H_2/CO_2 ratio (230 °C, 25 bar). **d** Dependence of CO yield on temperature and H_2/CO_2 ratio (1 bar). The thermodynamic calculation is a minimization of the Gibbs free energy of a system of pre-specified products (CH_3OH, DME, and CO) and reactants.

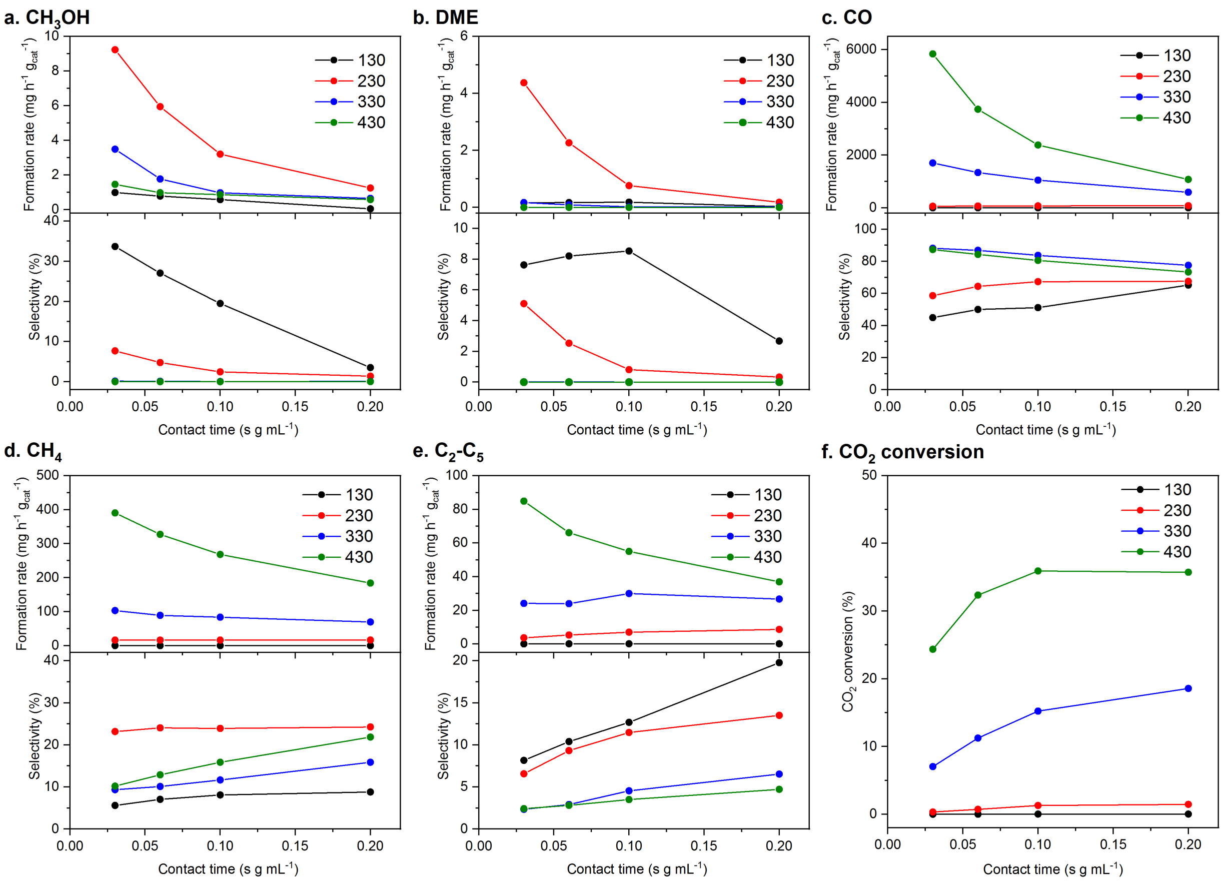

Supplementary Fig. 37. Catalytic performance of Mo_2CT_x at different temperatures. Reaction conditions: 130–430 °C, 25 bar, $H_2/CO_2/N_2$ = 3/1/1.

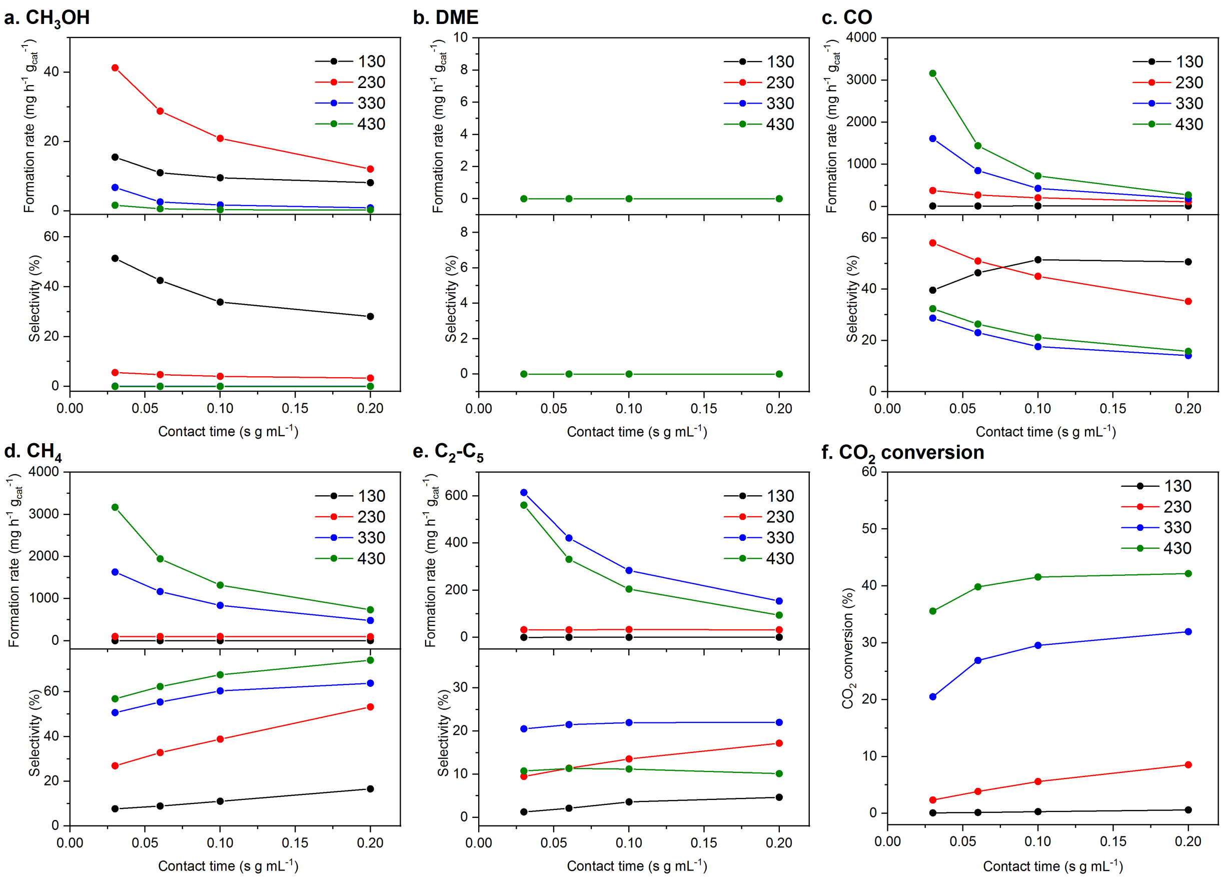

Supplementary Fig. 38. Catalytic performance of 2D-Mo_2C at different temperatures. Reaction conditions: 130–430 °C, 25 bar, $H_2/CO_2/N_2$ = 3/1/1. Note: no DME is detected at all of the tested temperatures leading to overlapping lines.

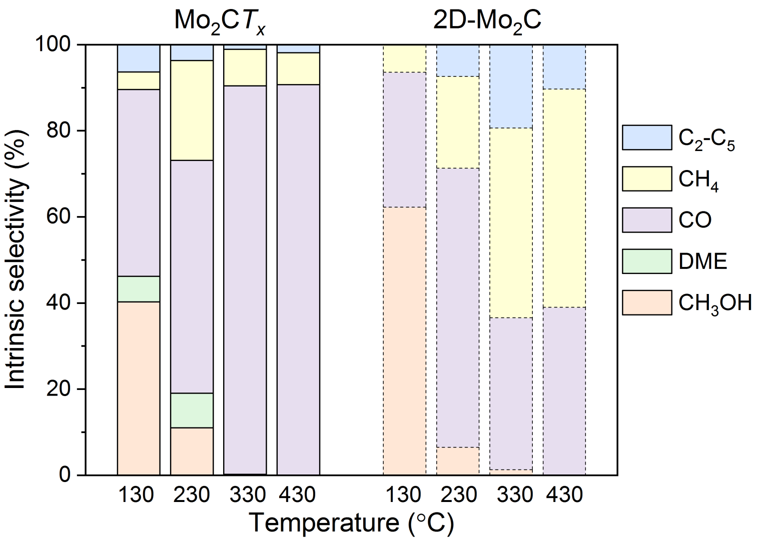

Supplementary Fig. 39. Intrinsic selectivity at different reaction temperatures. Reaction conditions: 130–430 °C, 25 bar, $H_2/CO_2/N_2$ = 3/1/1. Intrinsic selectivities were calculated from the intrinsic formation rates of the products.

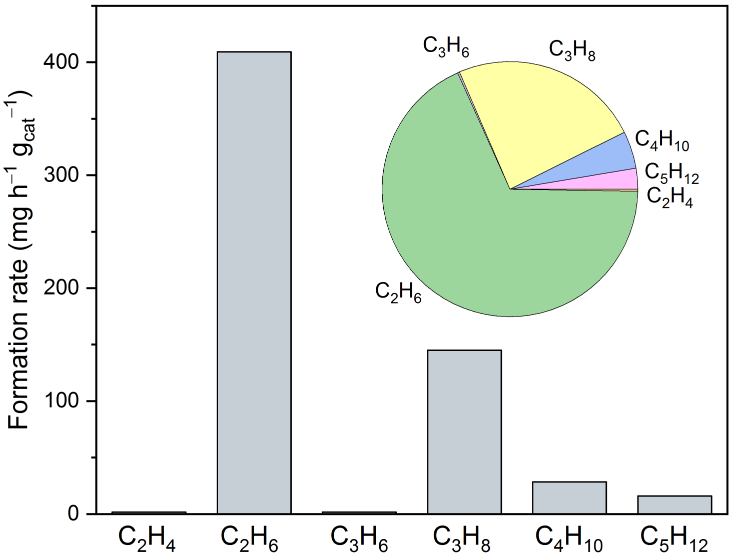

Supplementary Fig. 40. C_2–C_5 hydrocarbons generated from CO_2 hydrogenation using a 2D-Mo_2C catalyst (330 °C, 25 bar, $H_2/CO_2/N_2$ = 3/1/1, contact time 0.03 s g_{cat} mL^{-1}).

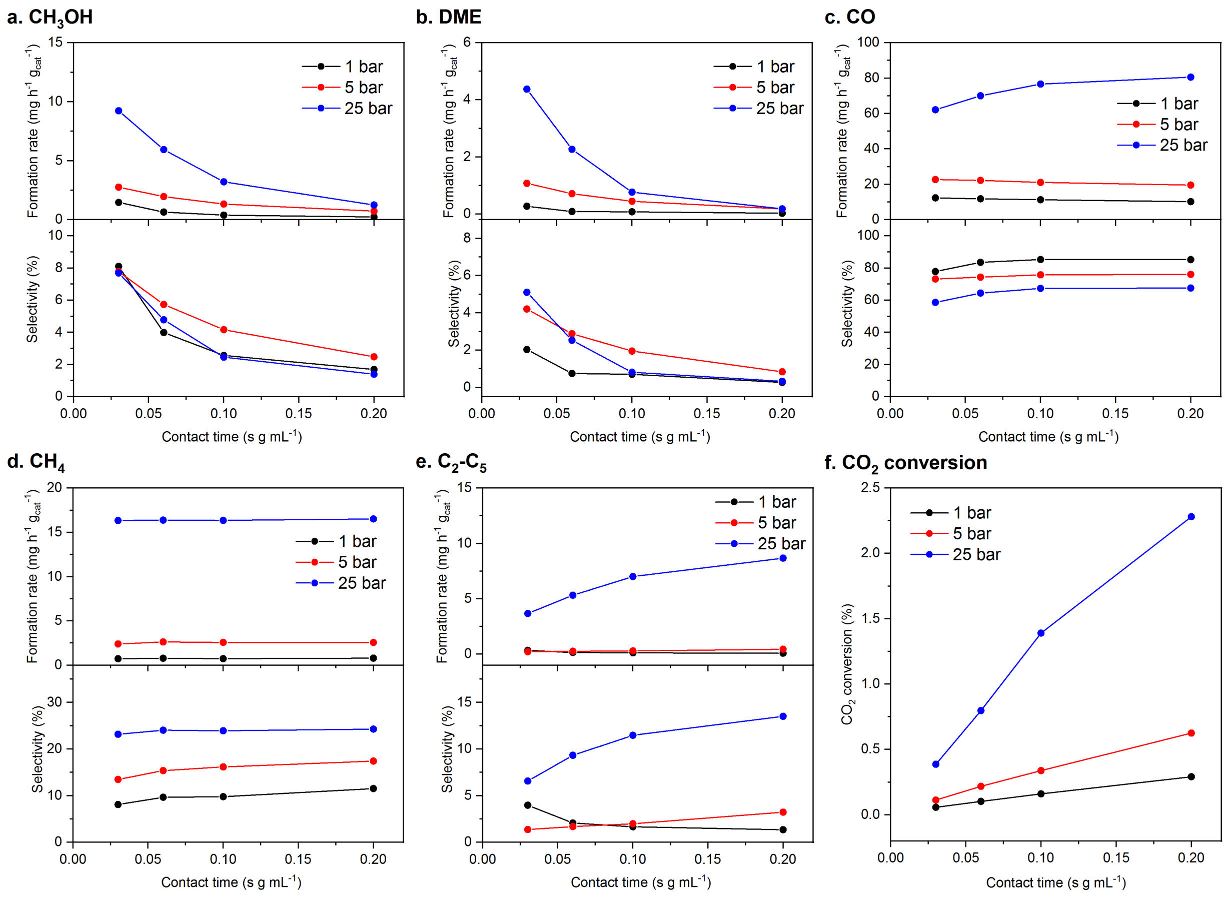

Supplementary Fig. 41. Catalytic performance of Mo_2CT_x at different pressures. Reaction conditions: 230 °C, 1–25 bar, $H_2/CO_2/N_2$ = 3/1/1.

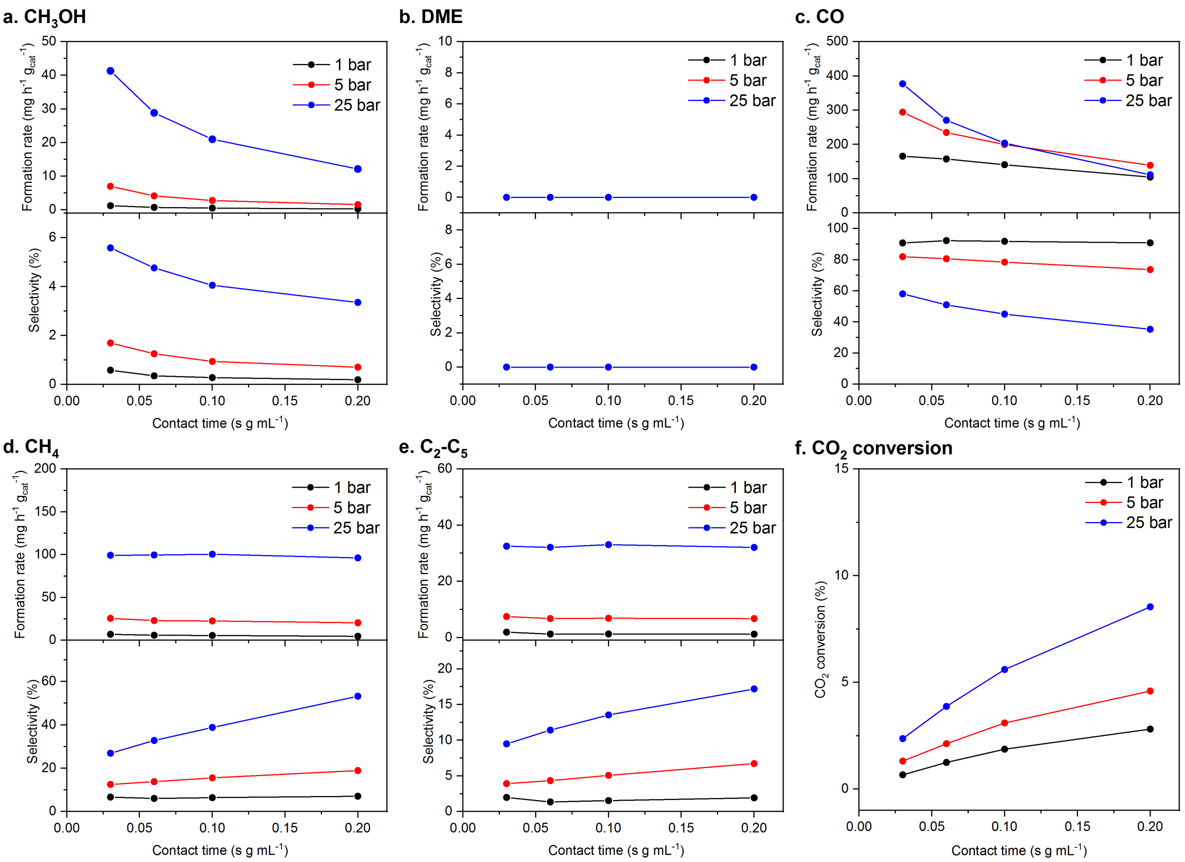

Supplementary Fig. 42. Catalytic performance of 2D-Mo_2C at different pressures. Reaction conditions: 230 °C, 1–25 bar, $H_2/CO_2/N_2$ = 3/1/1. Note: No DME is detected at all of the tested pressures yielding overlapping lines.

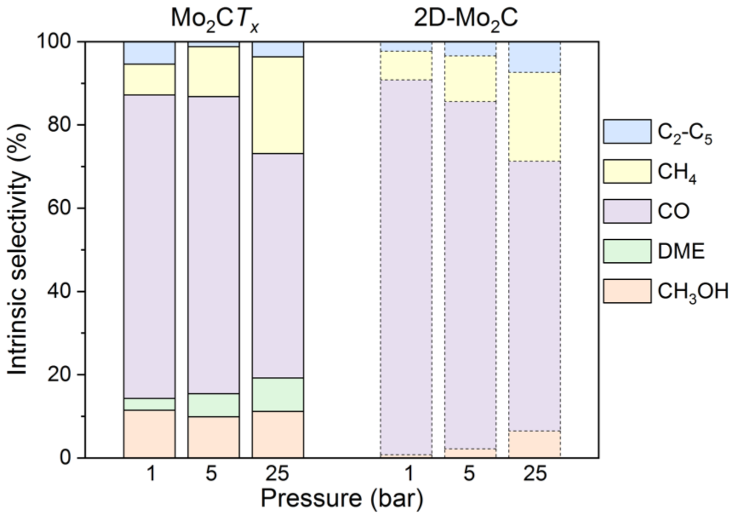

Supplementary Fig. 43. Intrinsic selectivity at different reaction pressures. Reaction conditions: 230 °C, 1–25 bar, $H_2/CO_2/N_2$ = 3/1/1. Intrinsic selectivities were calculated from the intrinsic formation rates of the products.

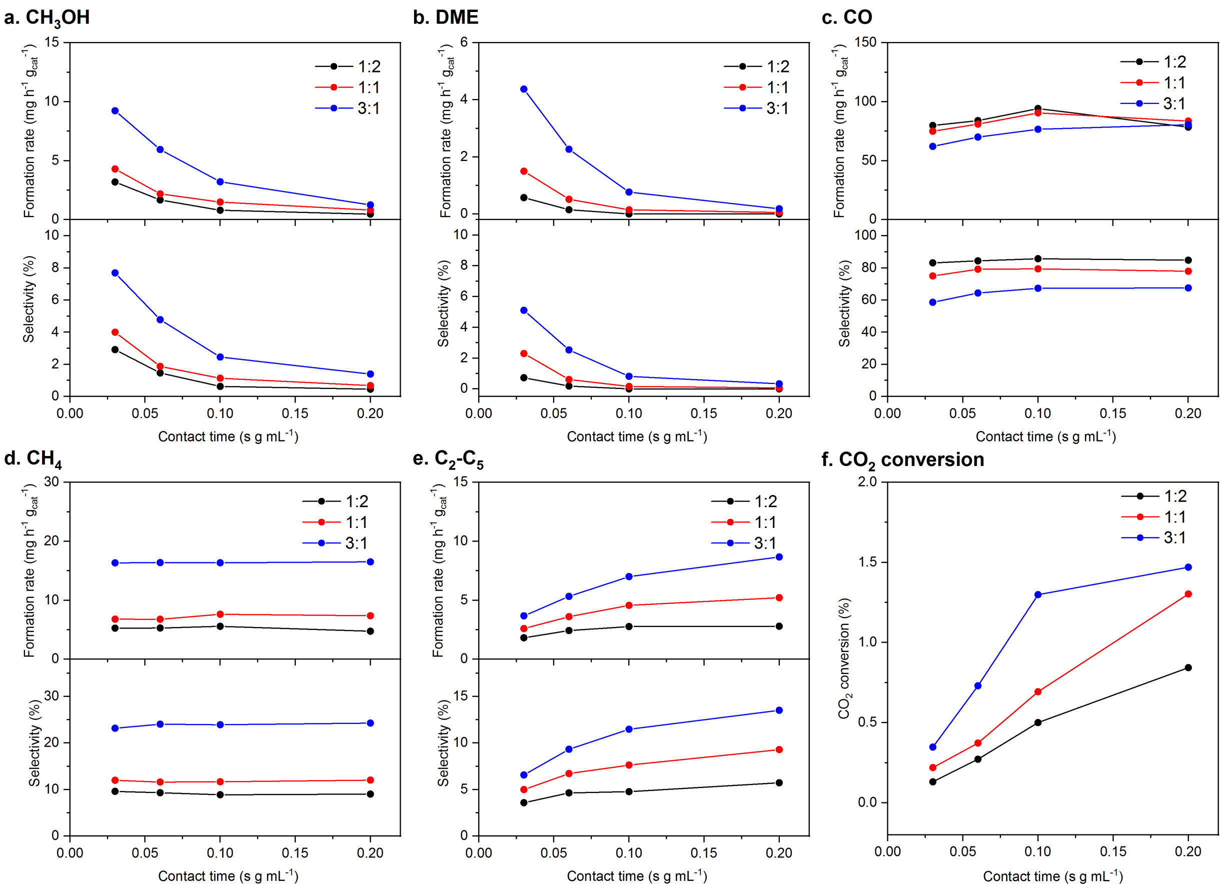

Supplementary Fig. 44. Catalytic performance of Mo_2CT_x with different H_2/CO_2 ratios. Reaction conditions: 230 °C, 25 bar.

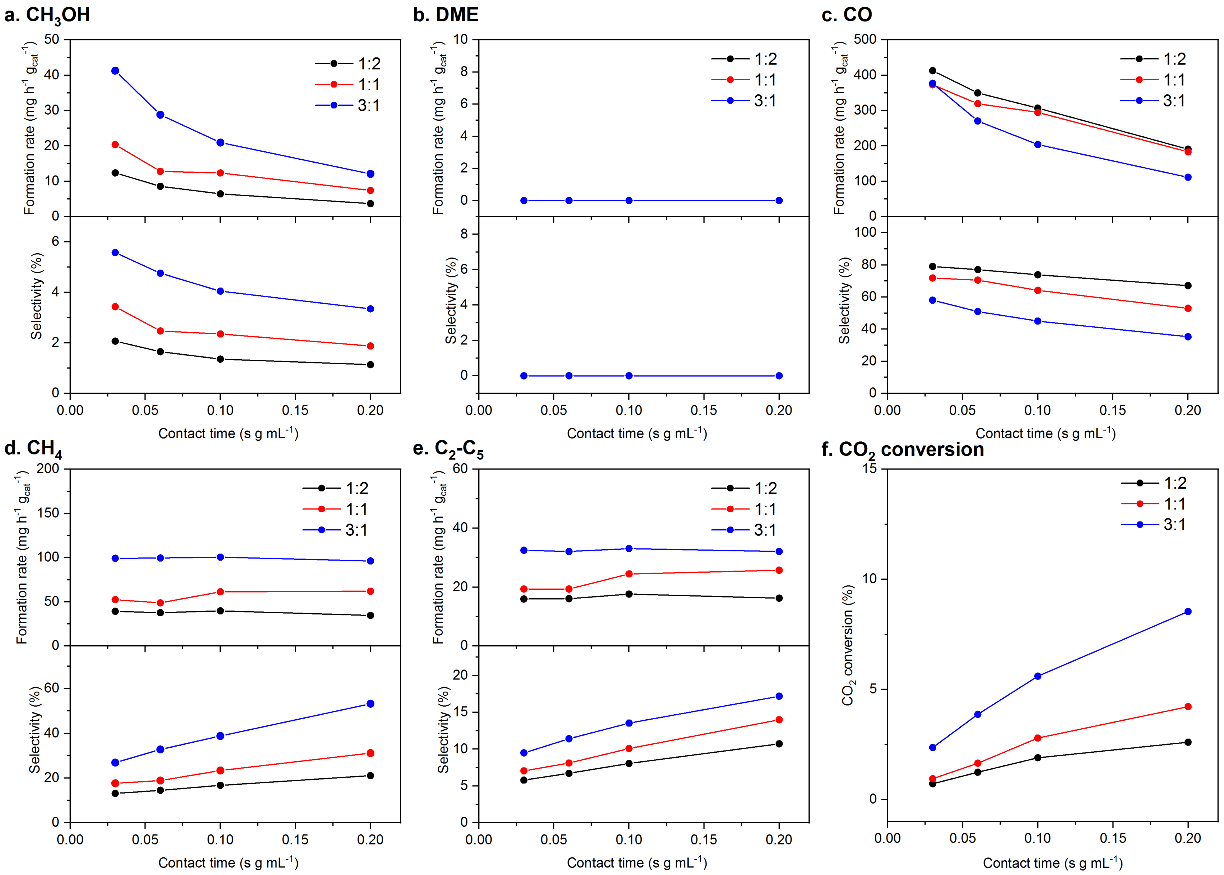

Supplementary Fig. 45. Catalytic performance of 2D-Mo_2C with different H_2/CO_2 ratios. Reaction conditions: 230 °C, 25 bar. Note: No DME is detected at all of the tested H_2/CO_2 ratios yielding overlapping lines.

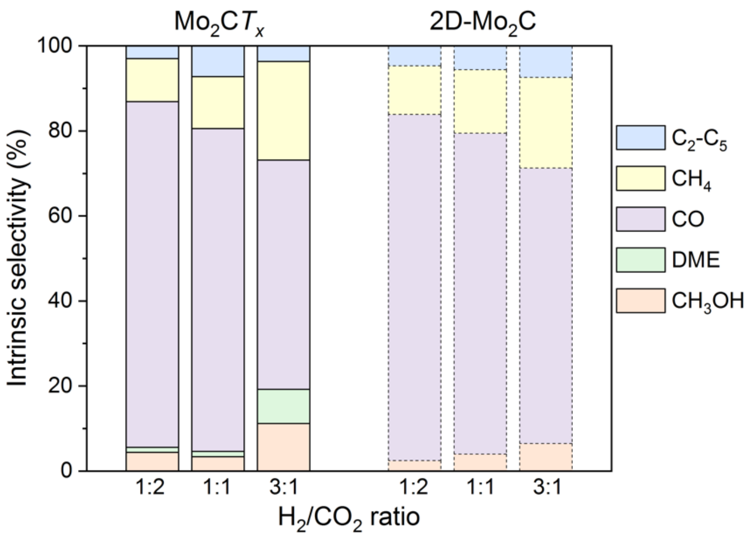

Supplementary Fig. 46. Intrinsic selectivity with different H_2/CO_2 ratios. Reaction conditions: 230 °C, 25 bar. Intrinsic selectivities were calculated from the intrinsic formation rates of the products.

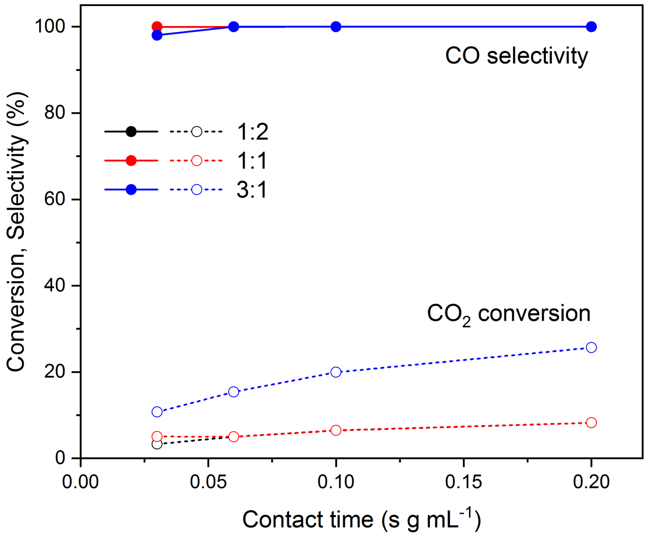

Supplementary Fig. 47. Reaction at 430 °C and 1 bar for high CO selectivity with Mo_2CT_x.

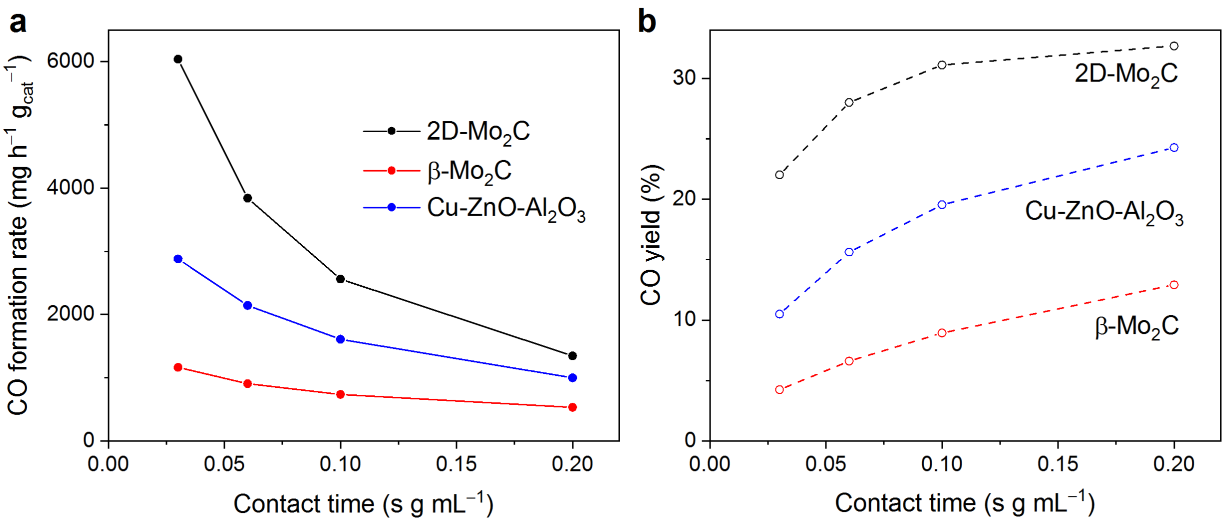

Supplementary Fig. 48. Comparison of CO formation rate and CO yield with 2D-Mo_2C, Cu-ZnO-Al_2O_3, and β-Mo_2C. **a** CO formation rate. **b** CO yield. Reaction conditions: 430 °C, 1 bar, $H_2/CO_2/N_2$ = 3/1/1.

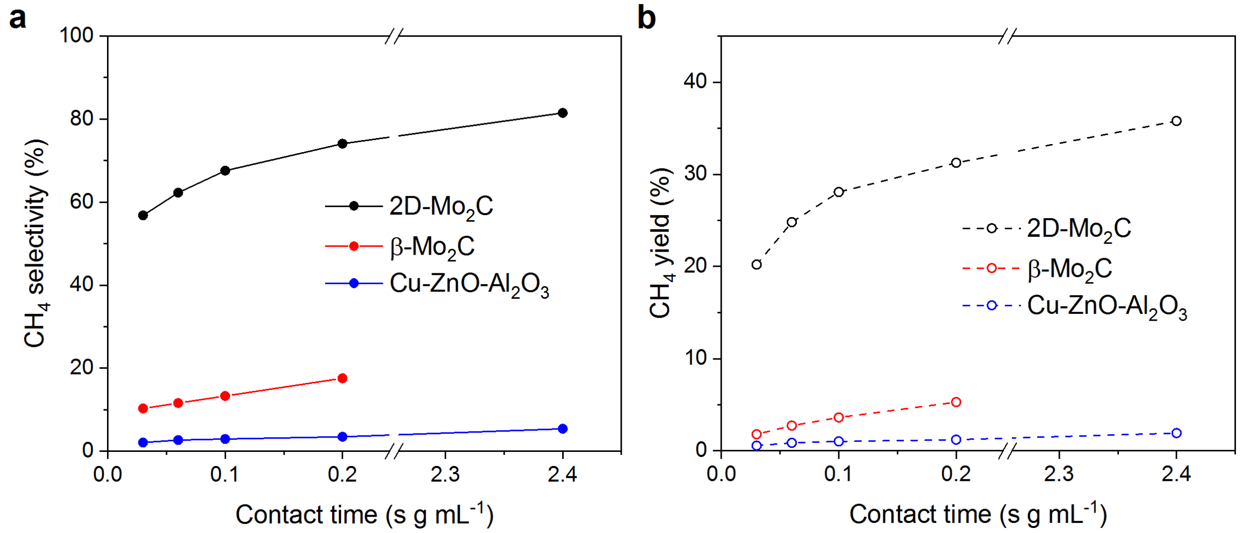

Supplementary Fig. 49. Comparison of CH_4 formation for 2D-Mo_2C, β-Mo_2C, and Cu-ZnO-Al_2O_3. **a** CH_4 selectivity. **b** CH_4 yield. Reaction conditions: 430 °C, 25 bar, $H_2/CO_2/N_2$ = 3/1/1.

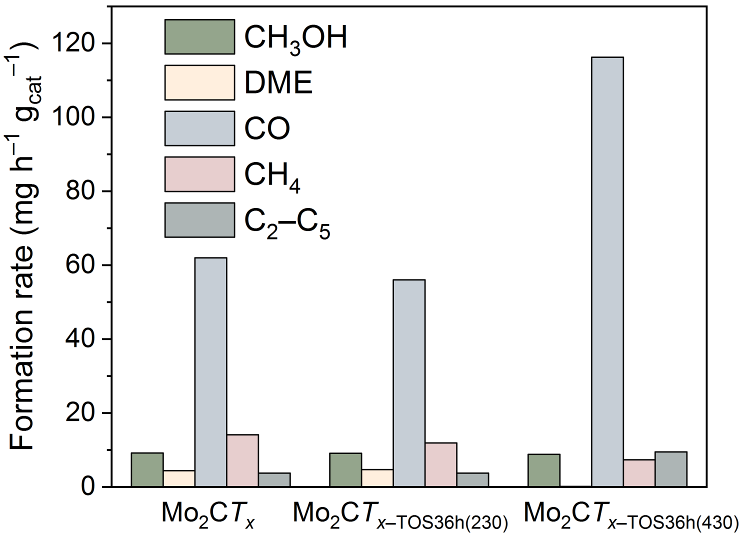

Supplementary Fig. 50. Stability of Mo_2CT_x during CO_2 hydrogenation reaction at standard conditions (230 °C, 25 bar, $H_2/CO_2/N_2$ = 3/1/1, contact time 0.03 s g mL^{-1}) after 36 hours of TOS at 230 and 430 °C, respectively.

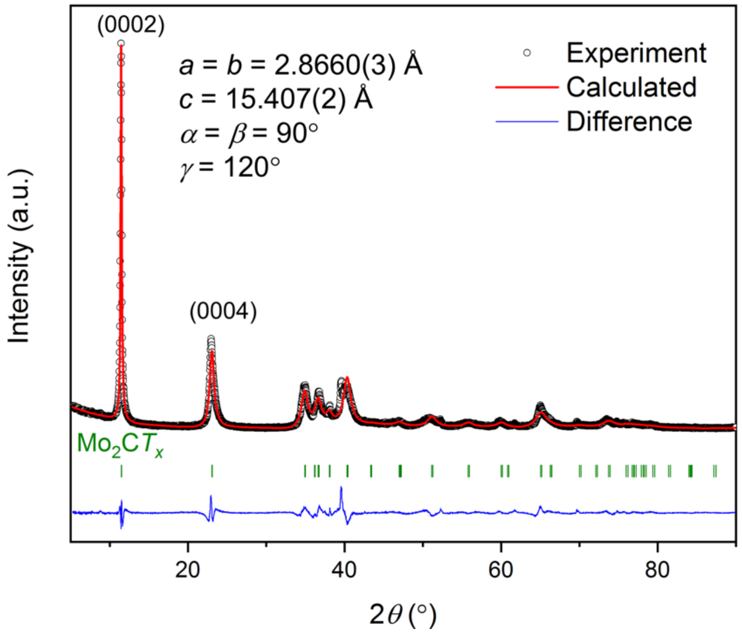

Supplementary Fig. 51. Le Bail fitting of the XRD pattern of Mo_2CT_x after 36 hours of TOS at 430 °C.

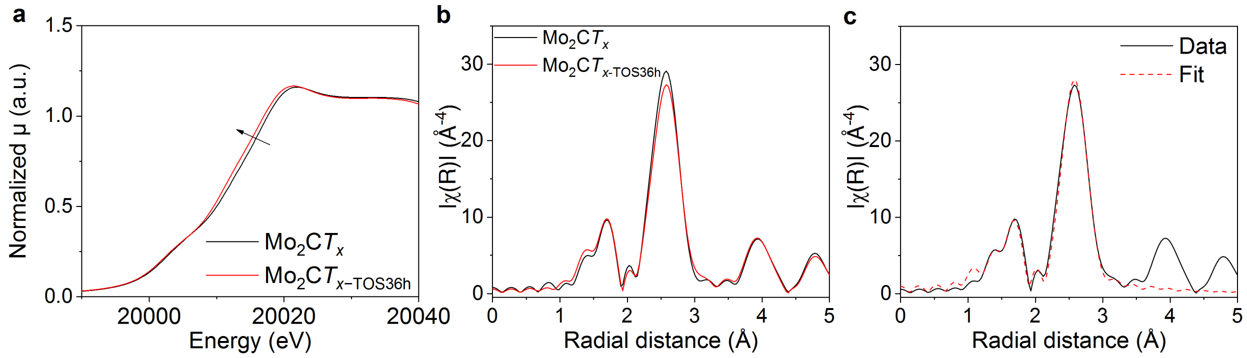

Supplementary Fig. 52. Mo K-edge XAS of Mo_2CT_x before and after 36 hours of TOS at 430 °C. **a** XANES. **b** EXAFS. **c** EXAFS fitting of $Mo_2CT_{x\text{-TOS36h}}$.

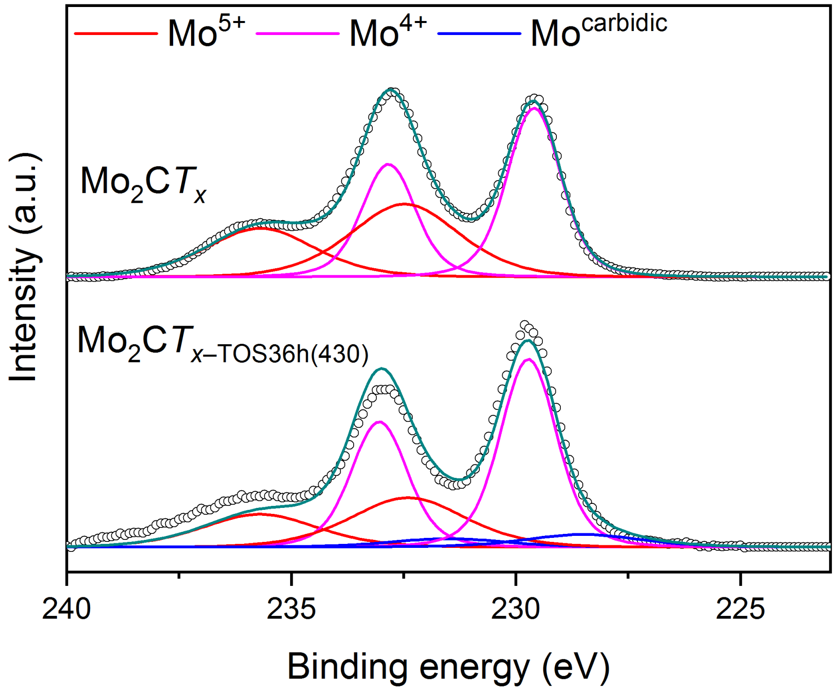

Supplementary Fig. 53. Mo 3*d* XPS of Mo_2CT_x before and after 36 hours of TOS at 430 °C.

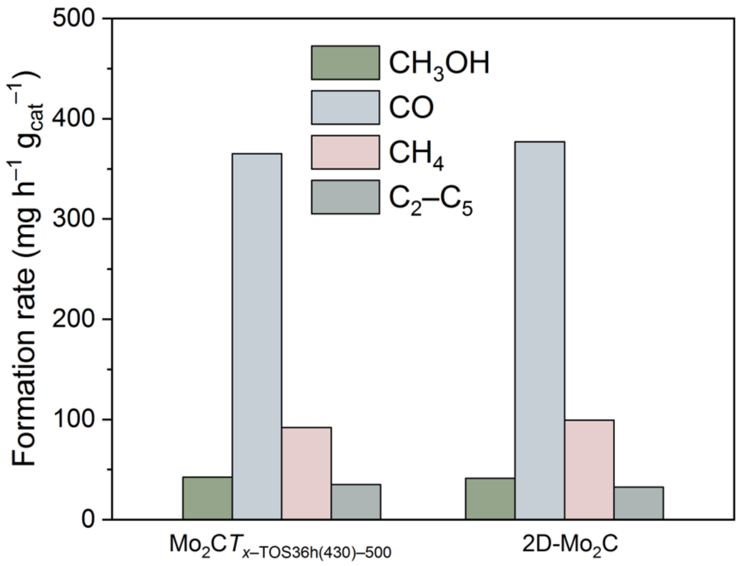

Supplementary Fig. 54. Comparison of Mo$_2$C$T_{x\text{-TOS36h(430)-500}}$ and 2D-Mo$_2$C. Reaction conditions: 230 °C, 25 bar, $H_2/CO_2/N_2$ = 3/1/1, contact time 0.03 s g mL^{-1}. Mo$_2$C$T_{x\text{-TOS36h(430)-500}}$ is Mo$_2$CT_x after 36 hours of TOS at 430 °C and 2 hours of reduction under 100% H_2 at 500 °C.

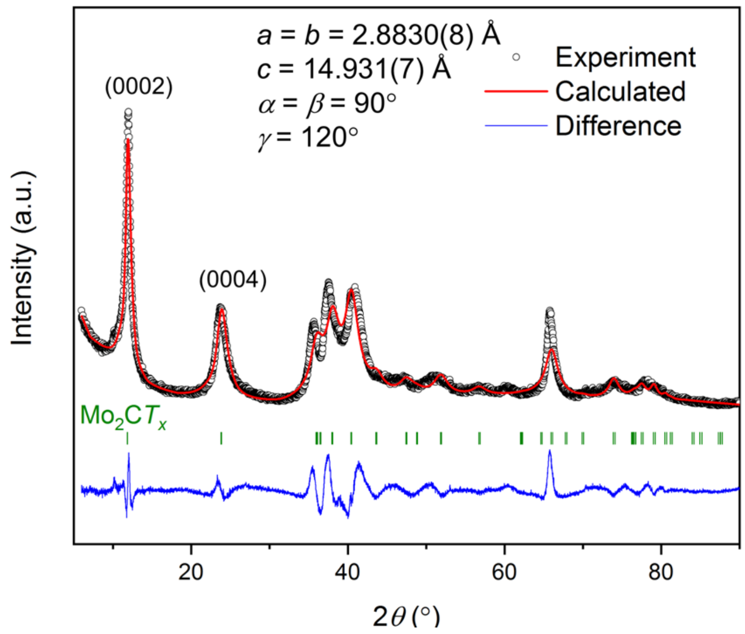

Supplementary Fig. 55. Le Bail fitting of the XRD pattern of 2D-Mo$_2$C after 36 hours of TOS at 430 °C.

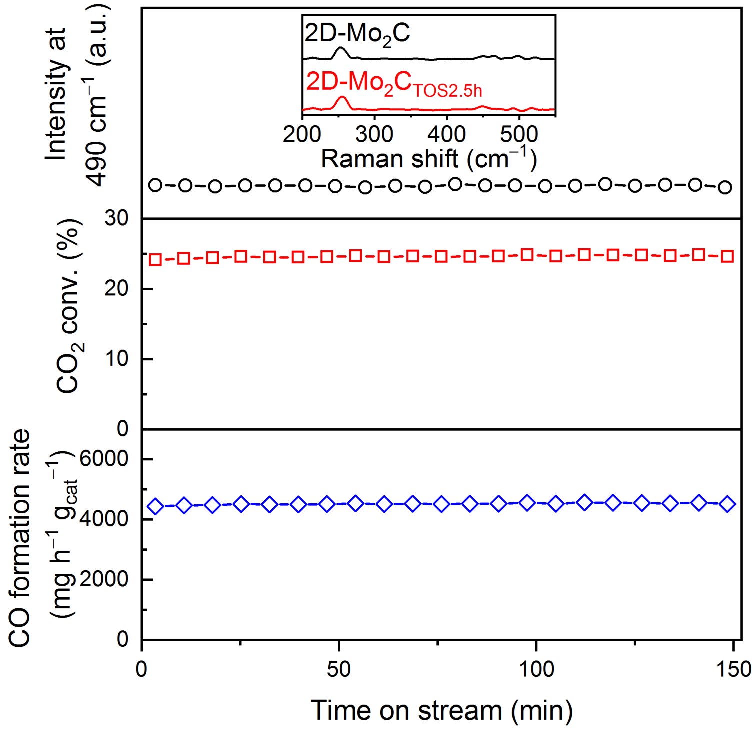

Supplementary Fig. 56. Operando Raman spectroscopy at CO_2 hydrogenation conditions with 2D-Mo_2C. Reaction conditions: 430 °C, 1 bar, $H_2/CO_2/N_2$ = 3/1/1.

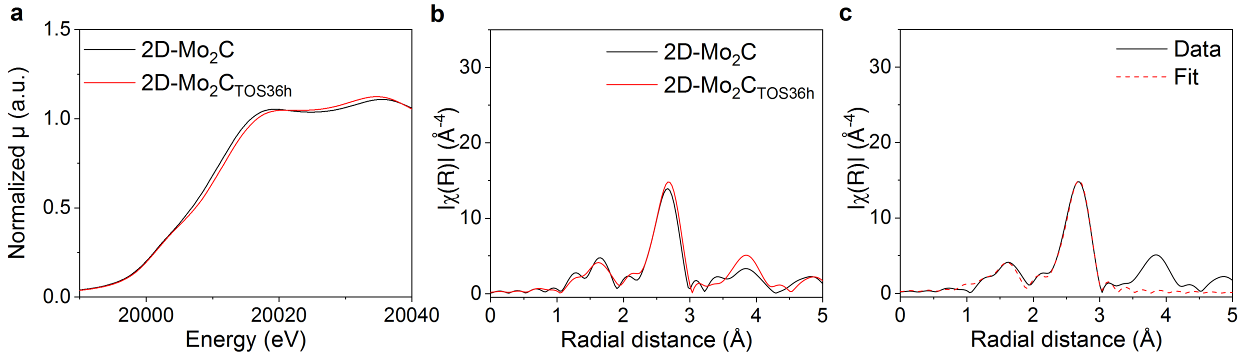

Supplementary Fig. 57. Mo K-edge XAS of 2D-Mo_2C before and after 36 hours of TOS at 430 °C. **a** XANES. **b** EXAFS. **c** EXAFS fitting of 2D-Mo_2C_{TOS36h}.

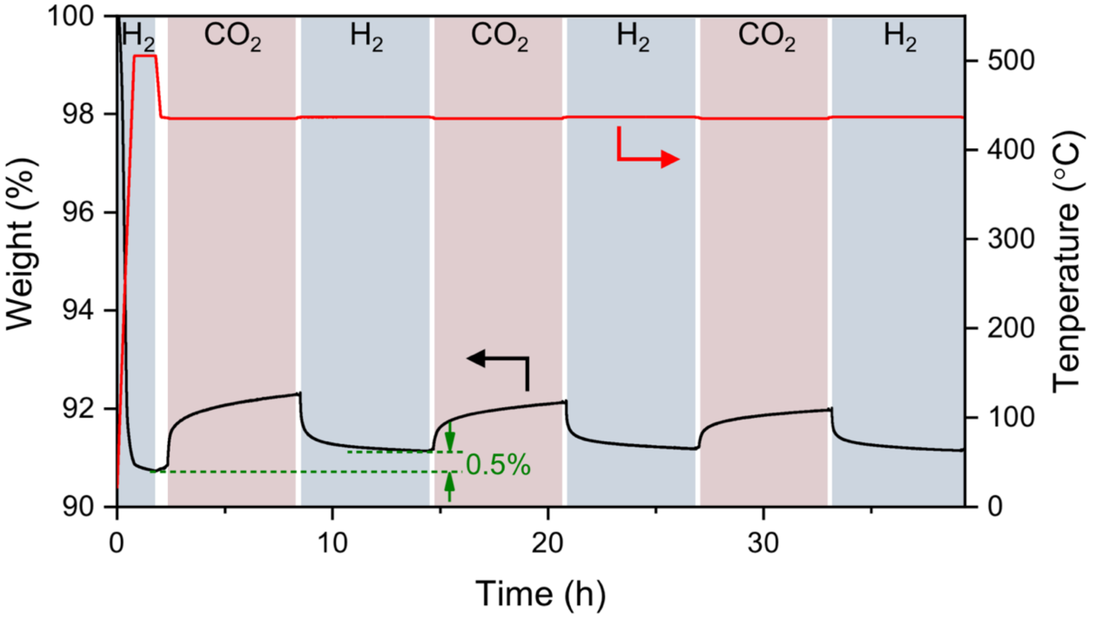

Supplementary Fig. 58. Chemical looping type operation of CO_2 hydrogenation studied by TGA. Sample amount: 55.104 mg Mo_2CT_x. The sample was first treated in 10% H_2/N_2 (100 mL min^{-1}) at 500 °C for 2 hours (5 °C min^{-1}). The flow was then alternated between 10% CO_2/N_2 (100 mL min^{-1}) and 10% H_2/N_2 (100 mL min^{-1}) using a pure N_2 purge for 10 min during the switch.

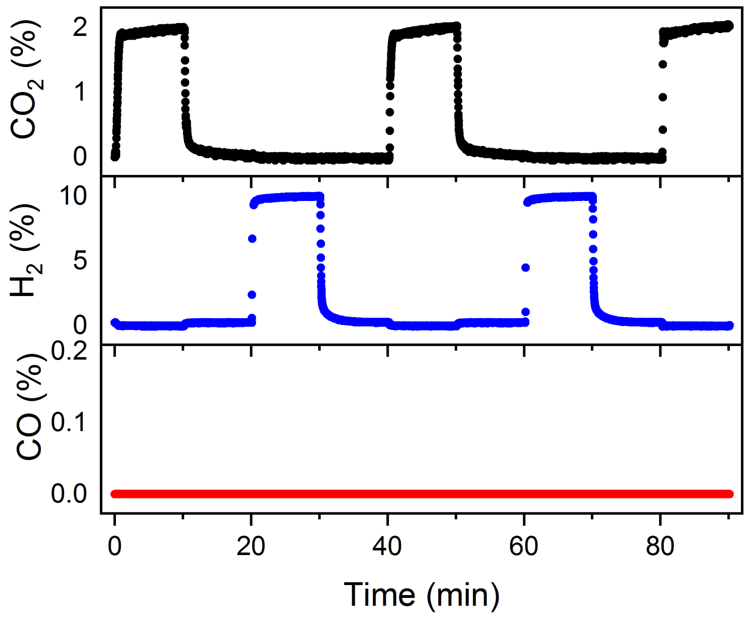

Supplementary Fig. 59. CO_2 dissociation study on β-Mo_2C (430 °C, 1 bar).

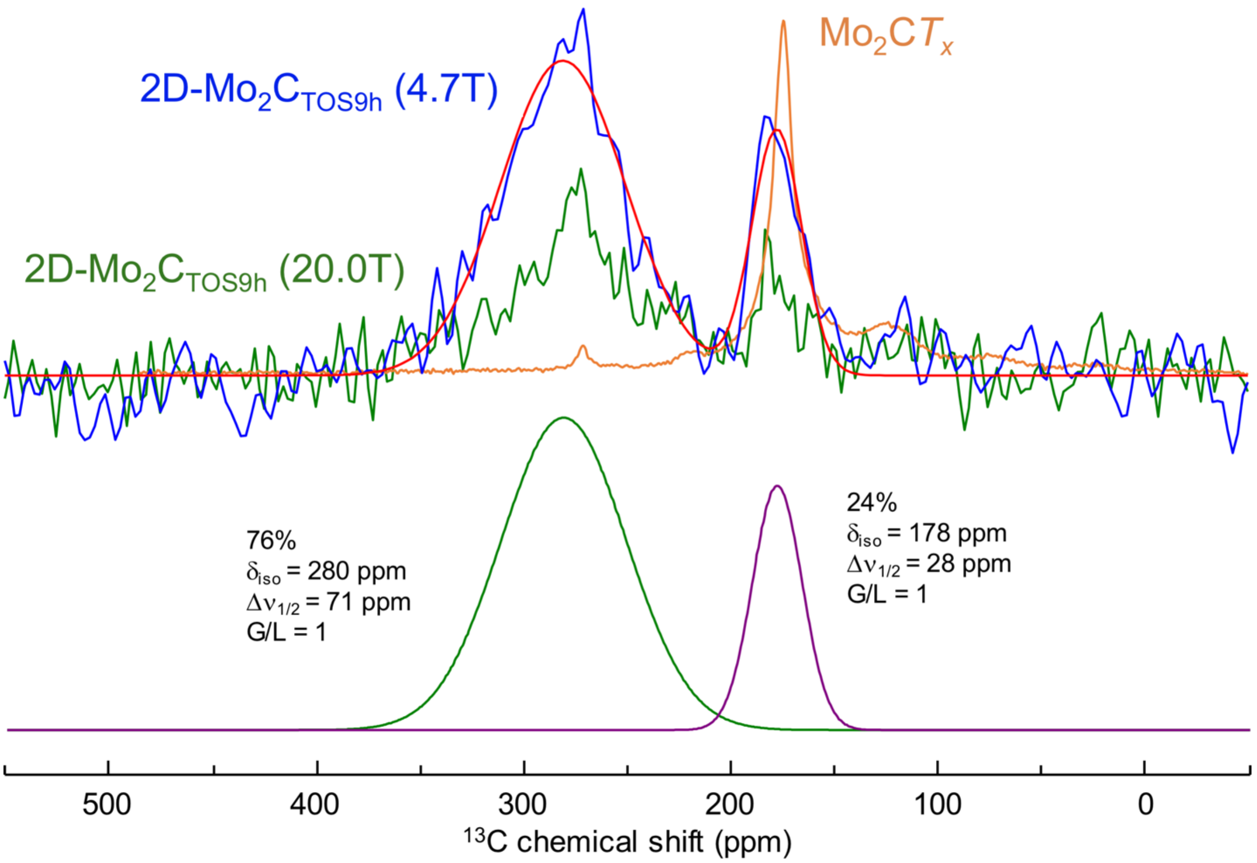

Supplementary Fig. 60. ^{13}C NMR of the 2D-Mo_2C after exposure to the reaction stream at 430 °C for 9 hours and the fresh Mo_2CT_x.

The ^{13}C spectra were obtained at a principal magnetic field of 4.7 T (i.e. a Larmor frequency of 50.3 MHz) using a 4 mm rotor diameter probe, spinning at 14.0 kHz. A Hahn Echo sequence was used, with a radio-frequency field of 52 kHz, a 90° pulse of 4.8 µs, a recycle delay of 2.5 s and 87000 accumulated scans. The additional 20.0 T spectrum was acquired under similar conditions: a 3.2mm rotor diameter probe spinning at 10.0 kHz, a radio-frequency field of 55.5 kHz, a "deteriorated" 90° pulse of 7.0 µs and a recycle delay of 1 s. The Mo_2C_{TOS9h} compound was handled in an argon-filled glove box and was mixed with BN to allow for spinning for which we used pure nitrogen. Spectra are referenced to tetramethylsilane (TMS).

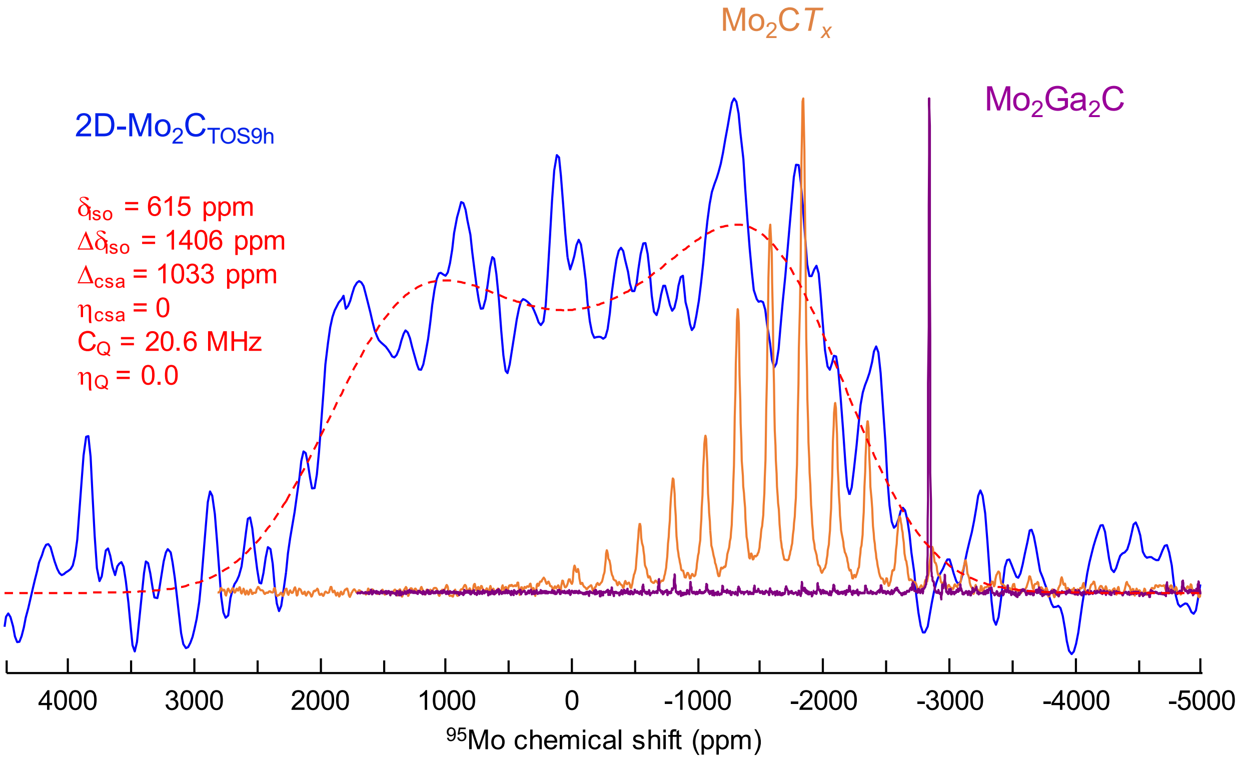

Supplementary Fig. 61. ^{95}Mo NMR of the 2D-Mo_2C after exposure to the reaction stream at 430 °C for 9 hours and the fresh Mo_2CT_x. Comparison with Mo_2Ga_2C is also given.

The ^{95}Mo spectra were obtained at a principal magnetic field of 20.0 T (i.e. a Larmor frequency of 55.1 MHz) using a 4 mm rotor diameter probe under static conditions and with a temperature regulation set at 5°C. A Hahn echo sequence was used to acquire each sub-spectrum of a VOCS procedure consisting of 21 spectra separated by an offset of 50 kHz. The radio-frequency field of 25 kHz, a 90° pulse of 5.0 µs and an echo delay of 150 µs was used. 14400 scans were acquired with a recycle delay of 0.5 s for each sub-spectrum. Spectra are referenced to a 2M solution of Na_2MoO_4.

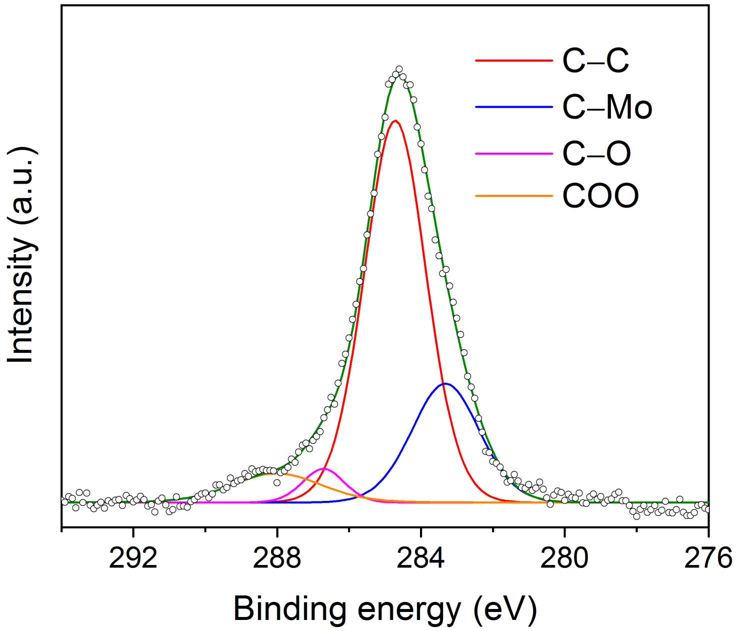

Supplementary Fig. 62. C 1*s* XPS of the Mo2C$T_{x\text{-}300}$ collected using an air-tight transfer cell. The peak assignment was performed following ref [2].

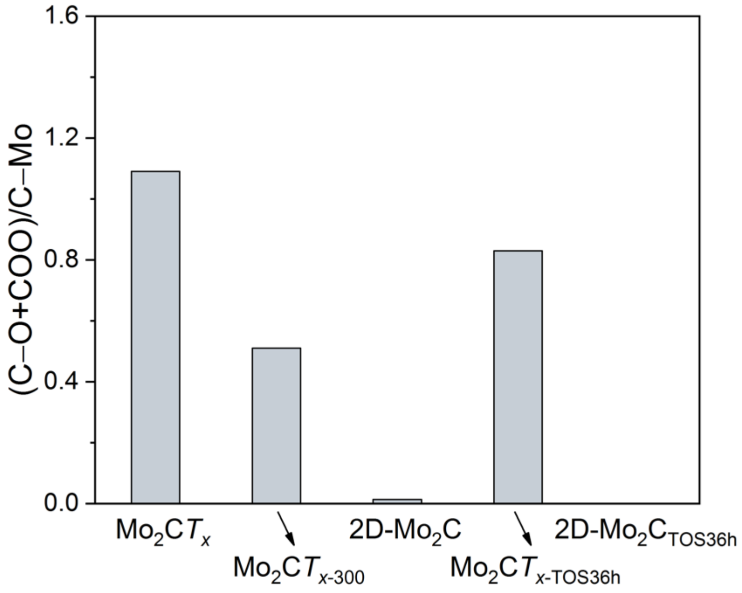

Supplementary Fig. 63. The (C–O+COO)/C–Mo ratio from C 1*s* XPS of the different catalysts assessed.

Supplementary Tables

Supplementary Table 1. XPS fitting parameters of Mo 3*d* for fresh and used (TOS = 36 hours) catalysts.

Material	State	Mo 3d 5/2 B.E. (eV)	ΔB.E. (eV)	FWHM (eV)	Peak shape	Amount (%)
β-Mo_2C	Carb.	228.5	3.1	1.3	LA[a]	100
Mo_2CT_x	Mo^{5+}	232.5	3.2	2.8	GL[b]	46
	Mo^{4+}	229.6	3.3	1.4	LA	54
Mo_2CT_{x-300}	Mo^{5+}	232.1	3.2	2.6	GL	26
	Mo^{4+}	229.8	3.2	1.6	LA	74
2D-Mo_2C	Carb.	228.4	3.2	1.5	LA	100
$Mo_2CT_{x-500-10\%}$	Mo^{4+}	229.3	3.2	1.9	LA	66
	Carb.	228.5	3.3	2.3	LA	34
$Mo_2CT_{x-TOS36h}$	Mo^{5+}	232.4	3.3	3.0	GL	31
	Mo^{4+}	229.7	3.3	1.5	LA	61
	Carb.	228.5	3.0	3.0	LA	8

[a]LA- Lorentzian Asymmetric line shape. [b]GL- Gaussian/Lorentzian line shape.

Supplementary Table 2. Mo K-edge EXAFS fitting of fresh and used (TOS = 36 hours) catalysts.

Material	Shell	CN	σ^2 (Å^2)	ΔE (eV)	R (Å)	R factor
β-Mo_2C	Mo-C	3*	0.004(1)	5.7(9)	2.09(1)	0.016
	Mo-Mo1	6*	0.006(1)		2.94(1)	
	Mo-Mo2	6*	0.006(1)		3.02(1)	
Mo_2CT_x	Mo-C/T_x	7(1)	0.005(1)	4(1)	2.10(1)	0.003
	Mo-Mo1	4.9(1)	0.003(1)		2.87(1)	
	Mo-Mo2	1.5(2)	0.003(1)		3.20(1)	
$Mo_2CT_{x\text{-}300}$	Mo-C/T_x	7(1)	0.005(1)	3(1)	2.11(1)	0.007
	Mo-Mo1	4.2(7)	0.003(1)		2.87(1)	
	Mo-Mo2	1.5(3)	0.003(1)		3.21(1)	
2D-Mo_2C	Mo-C/T_x	2.6(5)	0.003(1)	6(1)	2.09(1)	0.011
	Mo-Mo	6.1(7)	0.007(1)		2.95(1)	
$Mo_2CT_{x\text{-TOS36h}}$	Mo-C/T_x	7(1)	0.005(1)	4(1)	2.11(1)	0.006
	Mo-Mo1	4.9(8)	0.003(1)		2.87(1)	
	Mo-Mo2	1.8(3)	0.003(1)		3.21(1)	
2D-Mo_2C_{TOS36h}	Mo-C/T_x	3.2(7)	0.005(2)	4.8(9)	2.10(1)	0.010
	Mo-Mo	7.2(7)	0.007(1)		2.98(1)	

* Fixed CN. S_0^2= 0.96 was determined from the fitting of a Mo foil and fixed for all other samples.

Supplementary Table 3. Intrinsic activity of the catalysts for CO_2 hydrogenation (230 °C, 25 bar, $H_2/CO_2/N_2$ = 3/1/1).

Catalyst		β-Mo_2C	Mo_2CT_x	$Mo_2CT_{x\text{-}300}$	2D-Mo_2C	$Mo_2CT_{x\text{-}700}$
CO_2 reaction rate (mg h^{-1} g_{cat}^{-1})		144	164	96	1181	248
Product formation rate	CH_3OH (mg h^{-1} g_{cat}^{-1})	17	13	9	53	18
	DME (mg h^{-1} g_{cat}^{-1})	0	7	1	0	0
	CO (mg h^{-1} g_{cat}^{-1})	61	53	38	475	111
	CH_4 (mg h^{-1} g_{cat}^{-1})	6	16	5	98	12
	C_2–C_5 (mg h^{-1} g_{cat}^{-1})	2	2	3	32	5
Product selectivity	CH_3OH (%)	17	11	12	6	10
	DME (%)	0	8	2	0	0
	CO (%)	67	54	63	65	71
	CH_4 (%)	12	23	13	21	13
	C_2–C_5 (%)	5	4	9	7	6

Supplementary Table 4. CO generation from the CO_2 dissociation experiment.[a]

Cycle	1	2	3	4
CO amount (μmol g^{-1})	294	176	184	167

[a] Calculated based on the peak integration of Fig. 3a.

Supplementary References

1. Beale, A. M. & Sankar, G. In situ study of the formation of crystalline bismuth molybdate materials under hydrothermal conditions. *Chem. Mater.* **15**, 146–153 (2003).
2. Halim, J. *et al.* Synthesis and characterization of 2D molybdenum carbide (MXene). *Adv. Funct. Mater.* **26**, 3118–3127 (2016).
